# Metamaterials for Bone Repair: Design, Mechanisms, and Translation

**DOI:** 10.34133/bmr.0438

**Published:** 2026-11-13

**Authors:** Zhidong Liang, Jinbo Zhang, Xue Zhao, Le Gao, Mingju Yang, Zhaowei Zhang, Jincheng Wang, Hao Chen, Qing Han, Bingpeng Chen

**Affiliations:** ^1^ Department of Orthopedics, The Second Hospital of Jilin University, Changchun 130041, China.; ^2^ Department of Endocrinology and Metabolism, The First Hospital of Jilin University, Changchun 130021, China.

## Abstract

Large bone defects require scaffolds that combine mechanical support, biological activity, and anatomical adaptability. Metamaterials use deliberately designed structure to control effective properties beyond bulk composition. This narrative review examines metallic, ceramic, bioglass, polymeric, and composite metamaterial platforms for bone repair. It then considers mechanical, electroactive, magnetic, ultrasound-responsive, photoresponsive, thermoresponsive, and shape memory strategies. These structures can regulate modulus, stress transfer, pore connectivity, mass transport, degradation, and local stimulus delivery. The resulting local conditions influence bone ingrowth, vascularization, osteoimmune transitions, infection control, posttumor reconstruction, and osseointegration. At the cell–material interface, structural and physical influence engage yes-associated protein/transcriptional coactivator with PDZ-binding motif, integrin/focal adhesion kinase, Wnt/β-catenin, hypoxia-inducible factor-1α/vascular endothelial growth factor, bone morphogenetic protein/Smad, phosphatidylinositol 3-kinase/Akt/mechanistic target of rapamycin, and mitogen-activated protein kinase signaling. Translation remains constrained by limited fatigue data, poorly resolved in vivo pathway cross-talk, manufacturing variability, and excessive system complexity. Future designs should match a defined clinical indication and prioritize scalable fabrication, predictable mechanical and degradation behavior, long-term remodeling, and clinically relevant validation.

## Introduction

Bone can heal without intervention, but critical-sized defects caused by trauma, infection, tumor resection, or congenital disease exceed this capacity and disrupt skeletal continuity [1]. These defects impair both load transfer and the biological environment required for regeneration. Autologous cancellous or vascularized grafting remains the clinical benchmark because it supplies viable cells, extracellular matrix (ECM), osteogenic signals, and structural support [2]. Its use is limited by graft volume, donor site morbidity, longer procedures, and infection risk [3]. These constraints have accelerated the development of engineered scaffolds that provide defect filling while directing mechanical stability, tissue invasion, and host remodeling.

Metals, ceramics, polymers, and composites provide different but incomplete solutions. Metallic implants deliver load-bearing strength, yet excessive stiffness can shield the surrounding bone. Calcium phosphate (CaP) ceramics reproduce mineral chemistry and support continuous bone ingrowth [4], but brittleness limits their use in demanding load-bearing sites. Polymers are processable and degradable, although they often lack sufficient stand-alone strength. Composite systems can combine these properties but introduce additional manufacturing and regulatory complexity. Across all platforms, reducing effective modulus can limit stress shielding, whereas excessive porosity can compromise structural stiffness [5]. The central design problem is therefore not porosity alone, but the coordinated control of stiffness, strength, permeability, degradation, and tissue integration.

Metamaterials address this problem through designed unit cells whose geometry and spatial arrangement govern macroscale behavior. In this review, a bone repair metamaterial is a scaffold in which the designed unit cell directly contributes to a defined mechanical or transport response. A scaffold is not called a metamaterial solely because it is porous or produced by additive manufacturing. Mechanical metamaterial concepts provide the engineering foundation [6,7]. As shown in Fig. 1, this narrative review organizes current evidence by material platform, functional strategy, regenerative mechanism, and translational requirement. Additive manufacturing enables patient-specific geometries, graded porosity, and multiscale interfaces. Diamond lattices can alter bone ingrowth [8], and trabecular-like architectures can reproduce permeability and shear stress environments, although structural irregularity may reduce fatigue reliability (Fig. 2A) [9]. Effective design must therefore coordinate load transfer, mass transport, manufacturability, and biological activation. Its central contribution is to connect material architecture and functional activation with the temporal osteoimmune and angiogenic mechanisms of repair, while framing their clinical value through indication-specific evidence and manufacturability.

**Fig. 1.**
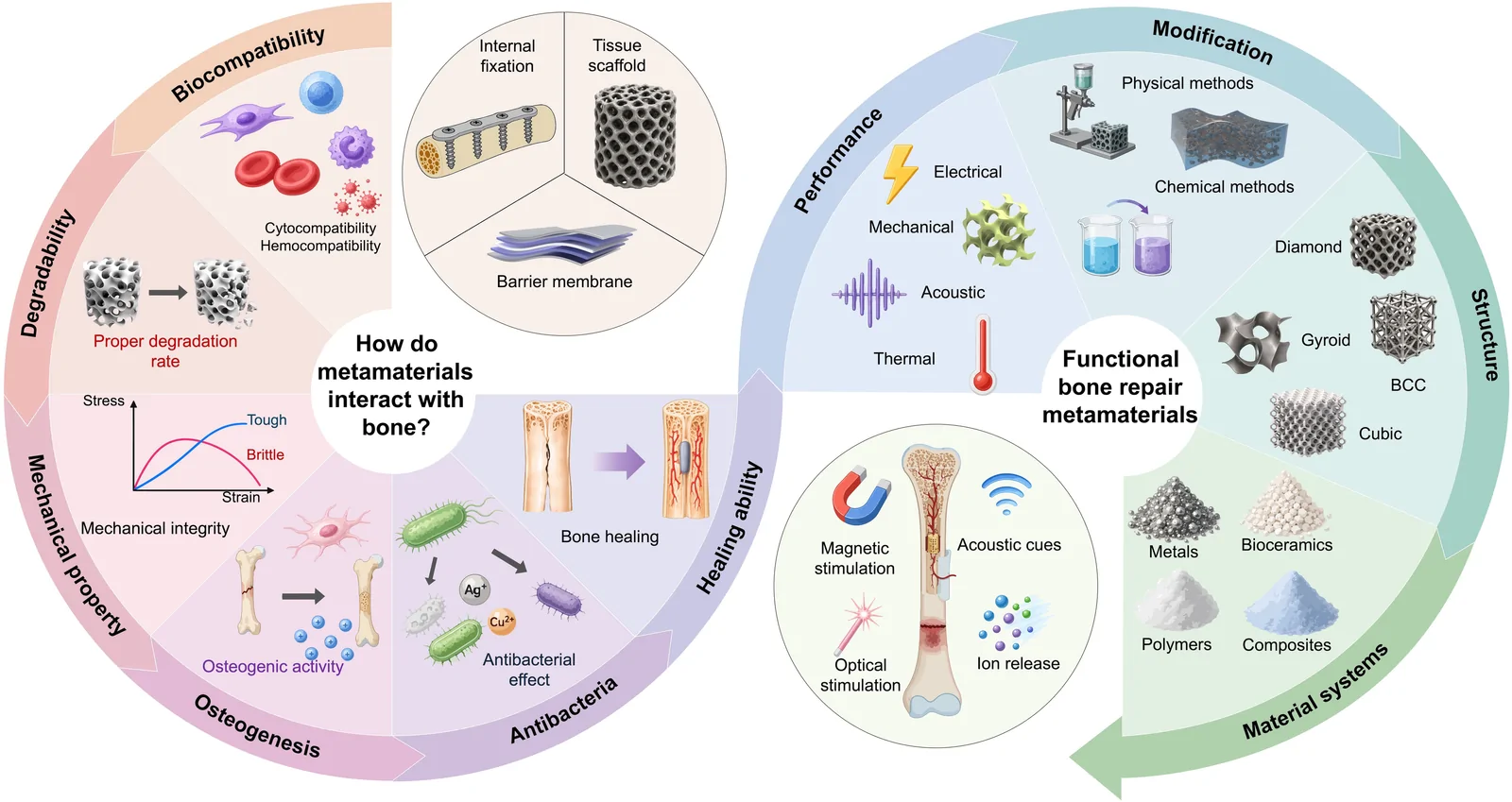
Schematic overview of the design logic and application framework of metamaterials for bone repair. Bone repair metamaterials are organized according to material composition, structural architecture, biological activity, and external physical responsiveness. BCC, body-centered cubic.

**Fig. 2.**
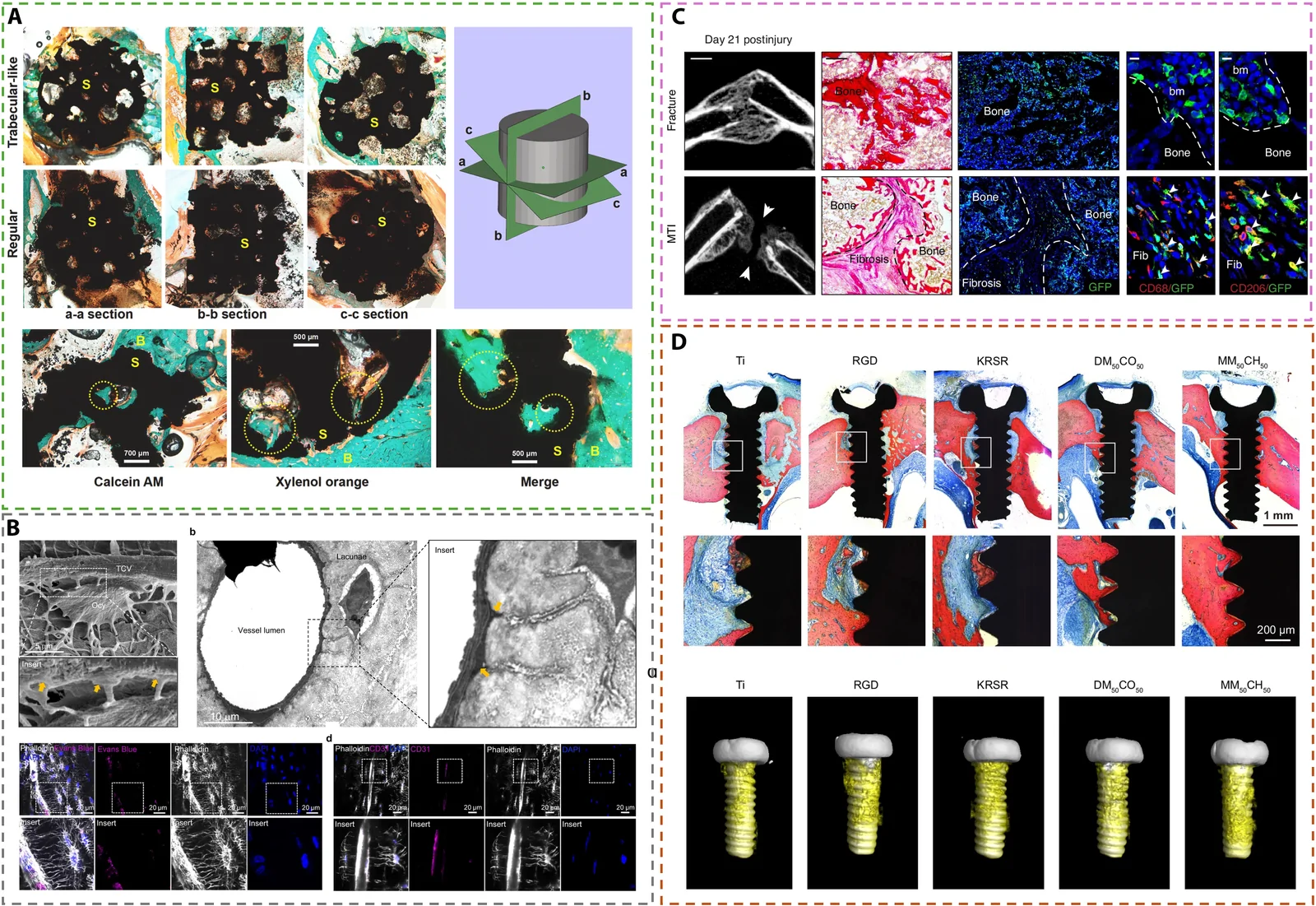
(A) Histological analysis of trabecular-like and regular scaffolds in rabbits 12 weeks after implantation. [9]. Copyright 2022, Elsevier. (B) Electron microscopy and fluorescence imaging showing the spatial relationships between osteocytes and transcortical vessels. [17]. Copyright 2024, Springer Nature. (C) Macrophage dynamics during bone repair revealed by multimodal immune-cell analysis [44]. Copyright 2024, Springer Nature. Scale bars, 1 mm (micro-CT and low-magnification images) and 10 μm (high-magnification immunofluorescence images). (D) Osteoblast-selective β-amino-acid polymer modification promoting implant osseointegration [56]. Copyright 2025, Springer Nature. DAPI, 4′,6-diamidino-2-phenylindole; GFP, green fluorescent protein; AM, acetoxymethyl; TCV, transcortical vessel; Ocy, osteocyte; bm, bone marrow; MTI, musculoskeletal traumatic injury; RGD, arginine–glycine–aspartic acid; KRSR, lysine–arginine–serine–arginine. DM_50_CO_50_ and MM_50_CH_50_ are β-amino acid polymer designations. DM_50_CO_50_ contains 50% dimethyl-substituted cationic subunits and 50% cyclooctyl hydrophobic subunits, whereas MM_50_CH_50_ contains 50% monomethyl-substituted cationic subunits and 50% cyclohexyl hydrophobic subunits.

## Bone Repair Biology

### Bone architecture and cells

Bone function depends on the coordinated organization of mineralized architecture, vascular networks, and specialized skeletal cells. Cortical and cancellous compartments provide distinct mechanical and transport environments, while osteoblasts, osteoclasts, osteocytes, and endothelial cells continuously remodel these regions. Osteoblasts produce and mineralize new matrix, whereas osteoclasts resorb aged or damaged bone. Osteocytes coordinate local mechanosensing and intercellular communication, and vascular networks provide oxygen, nutrients, and routes for progenitor recruitment. These processes are tightly coupled during bone homeostasis and defect repair. The anatomical organization of bone and the interactions among osteoblast-mediated bone formation, osteoclast-mediated resorption, and angiogenesis are illustrated in Fig. 3B. Understanding this structural and cellular integration provides the biological basis for designing metamaterials that regulate load transfer, mass transport, vascular ingrowth, and bone remodeling.

**Fig. 3.**
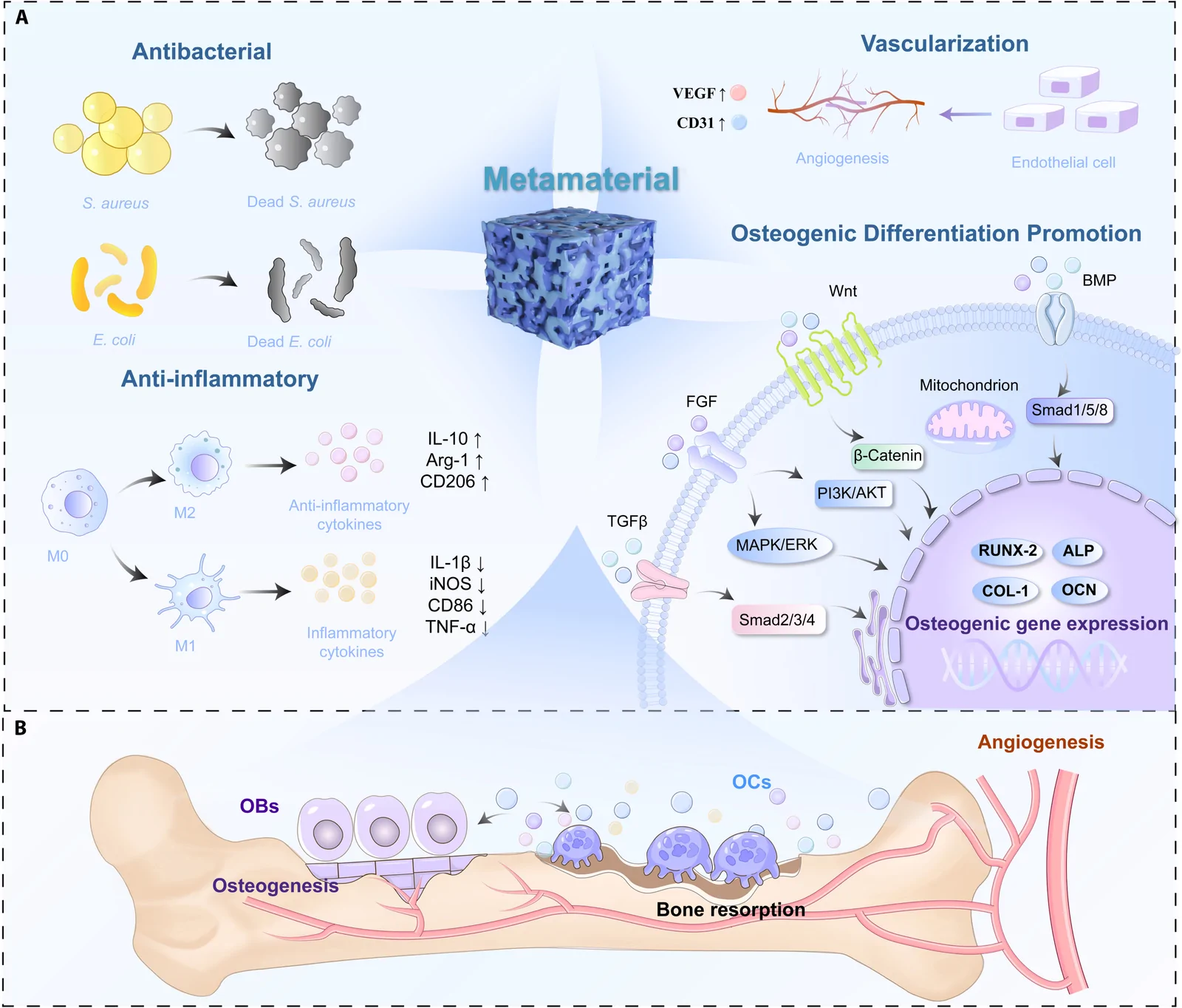
Biological functions of metamaterial scaffolds in bone repair. (A) After implantation, metamaterial scaffolds may regulate the local bone defect microenvironment through antibacterial effects, immune modulation, vascularization, and osteogenic differentiation. (B) Schematic representation of bone anatomy and bone remodeling, including osteoblast-driven bone formation, osteoclast-mediated bone resorption, and angiogenesis. IL-10, interleukin-10; iNOS, inducible nitric oxide synthase; TNF-α, tumor necrosis factor-α; FGF, fibroblast growth factor; Arg-1, arginase-1; OBs, osteoblasts; OCs, osteoclasts.

#### Bone architecture

Bone is a hierarchical organ comprising cortical and cancellous compartments, periosteum, endosteum, marrow, and interconnected cellular networks [10,11]. Cortical bone provides a dense load-bearing shell, whereas cancellous bone dissipates stress and supports marrow exchange. Their regional architecture distributes axial, bending, and torsional loads while preserving pathways for fluid and nutrient transport. Proximal femoral trabeculae form organized load-bearing trajectories rather than a random network [12], and cortical and cancellous regions differ markedly in tensile behavior [13]. These variations occur across multiple length scales, from organ-level geometry to lamellar organization. The resulting dense shell/porous core arrangement provides a design model for graded metamaterials that coordinate structural support, permeability, regional deformation, and tissue ingrowth.

#### Bone vasculature

Bone perfusion is supplied by nutrient, metaphyseal, epiphyseal, periosteal, endosteal, and marrow vessels. These networks support oxygen delivery, metabolic exchange, progenitor maintenance, and coupling between resorption and formation. The periosteum contributes both vascular support and progenitor populations, making its preservation important for early callus formation [11]. For scaffold design, the goal is not to reproduce every anatomical vessel but to create continuous, low-resistance pathways that permit vascular invasion from the defect margin to the core [14]. Channel orientation, interconnection, and maximum diffusion distance are therefore biologically relevant structural variables.

Intraosseous perfusion depends on connected marrow, endosteal, cortical, and periosteal networks [14,15]. Haversian and transverse channels distribute flow-through cortical tissue, while transcortical vessels link internal and external circulation [16]. Osteocyte-derived signals help maintain these channels and cortical angiogenesis [17] (Fig. 2B), demonstrating that vascular organization is actively regulated rather than anatomically fixed. Vascular access is therefore coupled to remodeling and should inform pore interconnectivity, channel orientation, surface curvature, and diffusion distance. Scaffolds that provide high total porosity but poorly connected central regions may still fail to support mature perfusion.

Neural and vascular regeneration are also coordinated during fracture repair [18]. Osteogenesis-associated vessels, including type H vessels, support callus consolidation and couple endothelial activity to osteoprogenitor function [19]. Proangiogenic factor delivery can accelerate vascular invasion when local retention and release are controlled [20]. Metamaterials should therefore provide not only osteoconductive surfaces but also spatial niches that support endothelial migration, perfusion recovery, and vascularized bone formation. This requirement becomes more important as defect size and the distance from viable host tissue increase.

#### Osteoblasts

Osteoblasts arise from skeletal mesenchymal progenitors and progress through lineage commitment, matrix production, and mineralization. Runt-related transcription factor 2 (RUNX2) controls early osteogenic fate and progenitor expansion [21,22], whereas epigenetic and mechanosensitive regulators influence commitment and the osteogenic–adipogenic balance [23–25]. Cell adhesion, cytoskeletal organization, and nuclear mechanics connect these programs to the extracellular environment. Consequently, scaffold stiffness, surface topography, pore wall curvature, and local strain can influence whether progenitors proliferate, remain undifferentiated, or enter the osteogenic lineage. These early responses determine the number and spatial distribution of matrix-producing cells within the scaffold.

Matrix maturation and mineralization are related but not synchronous. Transcriptomic analyses identify proliferative, matrix maturation, and mineralization phases [26], while transforming growth factor-β1 (TGFβ1) can restrain mineral deposition through the SMAD ubiquitination regulatory factor 1/CCAAT/enhancer-binding protein β/Dickkopf-related protein 1 axis [27]. Matrix vesicles create confined sites for CaP nucleation [28], and mitochondrial remodeling supports the energetic demands of osteogenic differentiation [29]. These processes link scaffold surface chemistry, ion release, pore-scale transport, and metabolic support to mineralized matrix formation. A scaffold that enhances early marker expression but limits oxygen, phosphate transport, or late-stage matrix organization may therefore produce incomplete regeneration.

Some mature osteoblasts become osteocytes and establish a 3-dimensional (3D) mechanosensory network. Osteocrin and Piezo1–yes-associated protein (YAP) signaling regulate dendrite formation and osteocyte maturation [30,31]. The continuity of this network is important for later load sensing and remodeling. Osteoblast-derived vesicles and apoptotic cell clearance also influence the transition from formation to resorption [32,33], whereas impaired RUNX2 stability can suppress osteogenesis [34]. Effective scaffolds should therefore support sustained mineralization, osteocyte embedding, dendritic connectivity, and later remodeling rather than only short-term increases in alkaline phosphatase (ALP) or osteogenic transcripts.

#### Osteoclasts

Osteoclasts derive from monocyte/macrophage precursors and differentiate under macrophage colony-stimulating factor and receptor activator of nuclear factor κβ ligand signaling [35]. Marrow adipogenic lineage precursors contribute RANKL during physiological and pathological remodeling [36], and mature osteoclasts can recycle through osteomorph intermediates [37]. Their abundance and activity vary across healing stages and disease states. Resorption is therefore a dynamic component of repair that prepares surfaces, removes low-quality tissue, and contributes to callus remodeling rather than a uniformly detrimental terminal event.

Mature osteoclasts require a sealing zone and ruffled border for matrix acidification and resorption. Hematopoietic protein-1 supports ruffled border formation [38], the galectin-3/low-density lipoprotein receptor-related protein 1 (LRP1) axis stabilizes cytoskeletal organization [39], and acidic conditions can enhance osteoclastogenesis through ovarian cancer G-protein-coupled receptor 1–Ca^2+^–calcineurin–nuclear factor of activated T cells signaling [40]. These processes depend on adhesion, polarization, local pH, and membrane trafficking. Scaffold degradation products, pore wall geometry, and surface chemistry may therefore alter osteoclast attachment and resorptive efficiency, with consequences for both material removal and host bone remodeling.

Osteoclasts also communicate with osteogenic cells. Secreted phosphoprotein 1-rich osteoclast extracellular vesicles can activate TGFβ1/SMAD3 signaling in mesenchymal cells [41], whereas mitochondria transferred from osteolineage cells can suppress myeloid resorption by altering redox metabolism [42]. This bidirectional coupling links resorption to subsequent matrix deposition and metabolic state. Metamaterial design should therefore coordinate osteoclast activity with osteoblast recruitment, scaffold degradation, and new bone replacement rather than indiscriminately suppress resorption throughout healing.

### Healing cascade

Fracture healing begins with hematoma formation, inflammation, and recruitment of skeletal progenitors [43]. Macrophage transitions, Tenascin-C-dependent mobilization, and mechanosensitive Periostin^+^ myeloid cells influence the early repair environment (Fig. 2C) [44–46]. These responses remove damaged tissue, regulate cytokine gradients, and prepare the defect for repair. Periosteal progenitors then contribute to fibrocartilaginous stabilization and early mechanical continuity [47,48]. Scaffold architecture and degradation should not prolong the inflammatory phase; instead, they should support timely immune resolution, progenitor entry, and provisional matrix formation.

Endochondral repair then replaces the cartilage template with woven bone. Chondrocyte hypertrophy, matrix mineralization, and vascular invasion must occur in a coordinated sequence. Hypertrophic chondrocytes support angiogenesis through thrombospondin-4 [49], while Piezo1-dependent mitochondrial regulation can promote the cartilage-to-bone transition through β-catenin/leucyl-tRNA synthetase 2 signaling [50]. Type H vessels and cysteine-rich angiogenic inducer 61-mediated angiogenesis further link perfusion recovery to ossification [19,20]. Structural mechanics, oxygen transport, and vascular support must therefore be coordinated during callus conversion, particularly in large defects where the central region is poorly perfused.

Remodeling converts woven bone into organized lamellar tissue through coupled osteoclast and osteoblast activity. Excess inflammatory osteoclasts can delay remodeling in osteoporotic fractures [51], and late repair includes cellular turnover, mineral redistribution, and maturation of tissue mechanics [52]. The scaffold should gradually transfer load to the regenerated tissue without leaving a mechanically weak interface or degrading before sufficient bridging has formed. Accordingly, bone repair metamaterials should regulate early immunity, progenitor recruitment, vascularized ossification, and degradation synchronized with host remodeling.

## Material Platforms

After implantation, adsorbed proteins and damage-associated signals initiate sterile inflammation and foreign body responses [53]. The composition and conformation of this protein layer determine how immune and skeletal cells first recognize the surface. Persistent inflammation and foreign body giant cell formation can promote fibrous encapsulation [54], whereas surface chemistry and topology also influence adaptive immunity [55]. Successful osseointegration therefore requires a controlled transition from inflammatory clearance to regeneration rather than complete suppression of inflammation. The major biological responses regulated by metamaterial scaffolds, including antibacterial activity, immune modulation, vascularization, and osteogenic differentiation, are summarized in Fig. 3A. Osteoblast-selective surface modification illustrates how interfacial design can bias cell recruitment and tissue integration toward bone formation (Fig. 2D) [56]. Material platforms should therefore be evaluated by both their bulk architecture and the host response generated at the pore wall.

Base materials define the feasible design space. Metals provide load-bearing strength and long-term interface stability [57]; ceramics and bioactive glasses support mineralization and ion-mediated signaling [58]; polymers provide conformability, degradation, and delivery capacity; and composites distribute these functions across phases [59]. Material selection also determines printing temperature, sterilization compatibility, achievable feature size, and the range of surface modifications that can be used. It therefore affects mechanics, degradation, host response, manufacturing reproducibility, and the incorporation of physical or biological stimuli.

### Metallic metamaterials

Metallic metamaterials use designed lattices to tune effective stiffness, stress transfer, permeability, and tissue-ingrowth pathways. An interconnected pore network can provide tissue access and mass transport without making a system a metamaterial. In this review, an architected scaffold has unit-cell geometry deliberately selected to generate a defined mechanical or transport response, whereas a porous scaffold is described by its void space without evidence that topology controls the reported function [60]. Additive manufacturing has enabled titanium and tantalum architectures with controlled porosity, curved surfaces, regional gradients, and patient-specific geometry [61,62]. The same architecture must survive cyclic loading while remaining open to tissue and vessel invasion. Translation therefore depends on reproducible printing, control of partially fused powder and surface defects, fatigue resistance, and reliable surface functionalization [63]. The term metamaterial is used below only when this structure–function link is explicit.

#### Titanium

Titanium and its alloys remain the most mature matrices for load-bearing orthopedic metamaterials because of their strength, corrosion resistance, and manufacturing experience. Dense titanium is substantially stiffer than bone and can redistribute load away from the host cortex. Indiscriminate porosity lowers effective modulus but can also reduce yield strength, fatigue life, and fixation reliability. Current designs therefore combine controlled topology, regional grading, and interfacial bioactivity rather than relying on random porous foams. The design objective is to preserve a safe load path while creating a connected biological space.

Titanium lattices have progressed from structural support to topology-guided regeneration. A 3D-printed titanium grid supported mandibular reconstruction in beagles and 3 patients without reported mechanical complications (Fig. 4C) [64]. This translational example shows the value of anatomical customization and stable fixation. Pei et al. [65] designed a homogeneous diamond-like titanium scaffold to mimic the osteocyte microenvironment and found that lattice geometry and implantation site jointly influenced in vivo bone regeneration. The study assessed osteocyte adhesion and migration in vitro and compared implantation in rabbit femur, beagle femur, and beagle skull sites in vivo. In controlled comparisons, diamond Ti6Al4V lattices combined a yield strength of about 140 MPa and an effective modulus of 11.6 GPa with enhanced cell adhesion, ALP activity, and mineralization [66]. Geometry therefore influences both mechanics and the cell-scale environment. These data argue against a universal optimal pore size; topology should be matched to anatomical loading, available host bone, vascular access, and biological transport.

**Fig. 4.**
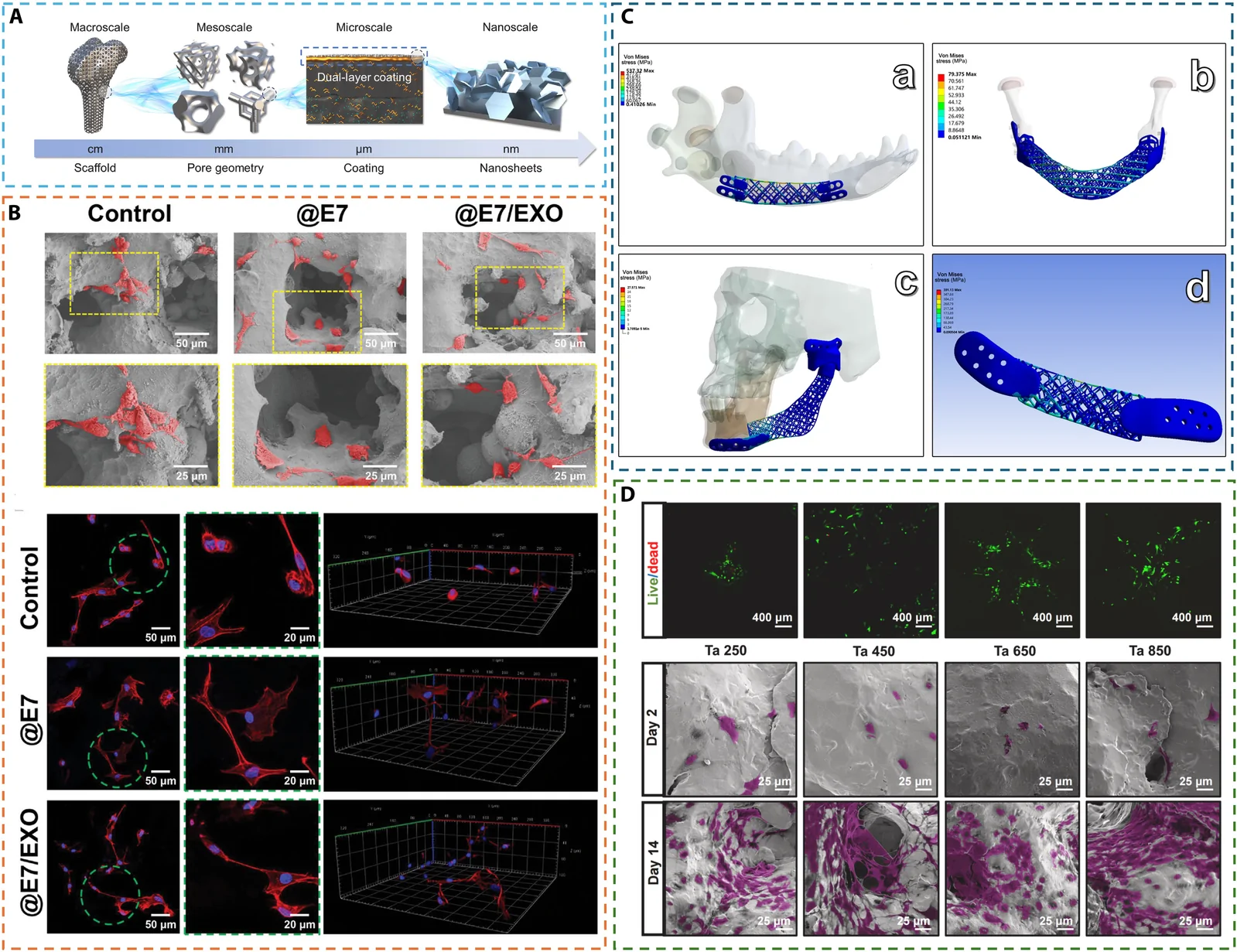
(A) Multiscale customized magnesium scaffold with hierarchical macro-, meso-, micro-, and nanoscale features for controlled degradation and bone repair [75]. Copyright 2024, Elsevier. (B) Cell adhesion and spreading on a functionalized 3D-printed Ti6Al4V “cell climbing frame” scaffold [69]. E7, E7 peptide; EXO, exosome. Copyright 2025, Wiley-VCH. (C) Finite element analysis of 3D-printed titanium grid scaffolds for mandibular segmental defect reconstruction [64]. Copyright 2023, Springer Nature. (D) Live/dead staining and osteogenic evaluation of porous tantalum scaffolds with different pore sizes [70]. Copyright 2024, Elsevier.

Functionally graded implants can distribute porosity according to regional mechanical and biological demands, and internal microporous zones have shown greater bone volume fractions [67]. These gradients can provide a denser fixation region and a more permeable regenerative core. Clinical studies of customized porous Ti6Al4V implants for large lower-limb defects also reported continued remodeling without scaffold fracture during intermediate follow-up [68]. Bioactive surface functionalization can further recruit and rejuvenate osteogenic cells (Fig. 4B) [69], but added coatings must remain stable during sterilization and implantation. Titanium platforms are therefore closest to clinical translation, yet long-term fatigue, large-animal weight-bearing validation, revision strategies, and reproducible multifunctional surfaces remain unresolved.

#### Tantalum

Tantalum offers high corrosion resistance, favorable interfacial bioactivity, and strong osteoconductivity. Its high melting point, density, cost, and processing difficulty historically limited geometrical control. Additive manufacturing now permits defined pore sizes and interconnected architectures, shifting tantalum from stochastic trabecular foams toward topology-controlled implants. This transition allows intrinsic material effects to be separated from the effects of porosity and geometry.

Pore architecture is a major determinant of tantalum osseointegration. In one specific study comparing scaffolds with nominal pore sizes of 250 to 850 μm, the 450-μm design showed the strongest bone response in both in vitro and large-animal experiments (Fig. 4D) [70]. This result, however, reflects the particular conditions of that study and should not be interpreted as a generally optimal pore size for bone regeneration. Interconnected macropores above approximately 300 μm have been widely investigated to support bone ingrowth and vascularization, and the appropriate pore size is likely to vary with material, porosity, geometry, loading conditions, and defect site [71]. Moreover, when pore size and porosity were matched, porous tantalum was more effective than titanium alloy in promoting osteogenic differentiation, mineralization, and regulation of inflammation [72]. Such structure-matched comparisons are especially valuable because they allow the contributions of material chemistry to be distinguished from those of pore architecture.

Current tantalum design increasingly couples architecture with disease-specific signaling. Spatially configured porous tantalum can activate the stromal-cell-derived factor 1α/CXCR4 axis and improve osteogenesis and vascularization in osteoporotic defects [73]. This approach extends tantalum from a passive osteoconductive matrix to an instructive interface. However, high cost, narrow processing windows, uncertain fatigue behavior, imaging artifacts, and limited manufacturing standards still restrict broad use. Its clinical value is therefore most likely in applications where rapid and durable osseointegration justifies the additional processing burden.

#### Degradable metals

Degradable metallic metamaterials must match transient mechanical support with corrosion and tissue replacement. Magnesium offers bone-like stiffness and osteogenic ion release but can corrode too rapidly, causing early loss of strength and local alkalization. Zinc degrades more slowly and avoids hydrogen evolution, but its lower strength constrains load-bearing use. Architecture and surface protection are therefore used to regulate exposed area, fluid access, ion release, and retained stiffness. Unlike permanent implants, these systems should be evaluated by the entire time-dependent trajectory of mechanics, degradation, and new bone formation.

Dicyclopentadiene-coated porous magnesium supported new bone formation while retaining about 52% of scaffold volume at 12 weeks [74]. A multiscale magnesium scaffold combined anatomical shaping, pore-curvature optimization, surface activation, and protective coatings to improve early support and degradation control (Fig. 4A) [75]. These studies show that corrosion cannot be managed by alloy composition alone. Bioactive coatings can support endothelial migration and vascularized repair, collagen layers can moderate Mg^2+^ release, and optimized WE43 printing and postprocessing can improve cytocompatibility and defect repair [76–78]. Zinc-based designs use alloying, gyroid architecture, surface nanopatterning, or exosome functionalization to balance strength, degradation, and osteoimmune activity [79–81]. Their slower corrosion may provide a wider therapeutic window, although long-term replacement and fatigue remain insufficiently defined. Beyond metallic systems, bioactive glass ceramic scaffolds have also been adapted for postosteosarcoma reconstruction (Fig. 5D) [82]. A 2-stage deformation architecture further showed that low effective modulus can coexist with load-bearing capacity and amplify callus strain, calcium signaling, and hypoxia-inducible factor-1α (HIF-1α)-associated angiogenesis [83]. These examples demonstrate that degradable-metal design is simultaneously a structural, electrochemical, and biological problem. Translation requires validated matching between corrosion, retained strength, ion exposure, vascularized tissue formation, and eventual scaffold replacement.

**Fig. 5.**
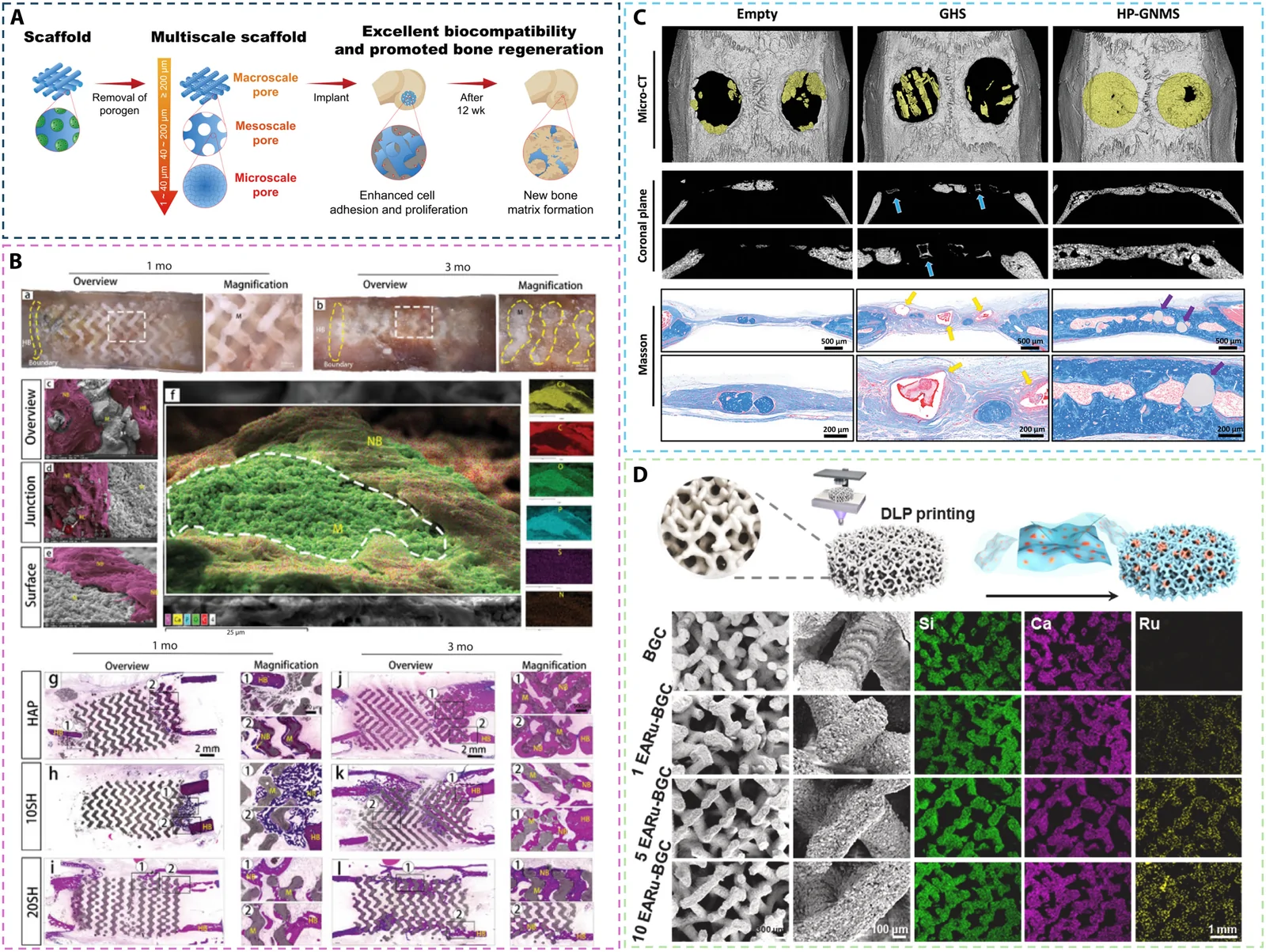
(A) Graphical summary of a multiscale 3D-printed silk porous hydrogel scaffold for enhanced cell infiltration and bone regeneration [111]. Copyright 2025, Elsevier. (B) In vivo evaluation of reinforced 3D-printed bioceramic scaffolds for supercritical bone defect reconstruction [92]. 10 SH, 10 wt% CaSO_4_ + HAP (hydroxyapatite); 20 SH, 20 wt% CaSO_4_ + HAP. Copyright 2024, Wiley-VCH. (C) Micro-computed tomography (CT) and histological evaluation of hierarchical porous nanofibrous scaffolds for bone regeneration [108]. Copyright 2024, Wiley-VCH. (D) Preparation and characterization of 2D nanosheet-modified 3D-printed bioactive glass ceramic scaffolds [82]. BGC, bioactive glass ceramic; EARu, ellagic acid–ruthenium; EARu-BGC, ellagic acid–ruthenium nanosheet-modified bioactive glass ceramic scaffold. Copyright 2025, Elsevier.

### Ceramic and bioglass metamaterials

Ceramic and bioglass metamaterials include hydroxyapatite, β-triCaP, biphasic CaP (BCP), mesoporous bioactive glass (MBG), and printable bone cements. Their mineral-like chemistry supports osteoconduction, while architected pores and micro/nanointerfaces improve fluid transport, cell distribution, and ion-mediated signaling [84,85]. Composition also determines dissolution kinetics and the release of calcium, phosphate, silicon, magnesium, or therapeutic dopants. Their main limitations remain brittleness, printing defects, and restricted long-term load-bearing capacity. Architecture can redistribute stress and improve permeability, but it does not eliminate the need to control fracture toughness and fatigue.

#### Calcium phosphates

Within CaP systems, 3D printing provides an important advance for achieving both macroscopic shape control and mesoscopic pore interconnectivity in brittle materials. Wang et al. [86] showed that BCP scaffolds with graded pores of 400 to 800 μm improved mass transport, cytocompatibility, vascularization, and new bone formation while maintaining 70% total porosity. This indicates that CaP scaffolds can regulate the repair microenvironment through spatially organized topology rather than acting only as mineral reservoirs. Qin et al. [87] further showed in a rabbit mandibular defect model that adjusting pore dimensions could alter bone repair outcomes, suggesting that pore topology is an important structural factor for vascular ingrowth and osseointegration in bioceramic systems. In addition to controlling pore architecture, Sun et al. [88] used controlled release of an osteogenic drug from a 3D-printed CaP scaffold, allowing local treatment to be coupled to a defined pore architecture. Zhang et al. [89] combined customized macroscopic shape with micronano inner porosity and surface design in printed bioceramics for bone regeneration.

Building on this topological foundation, Wang et al. [86] integrated triply periodic minimal surface (TPMS) gyroid architectures into BCP scaffolds and designed hierarchical gradients between peripheral and central structural zones. Their findings showed that a 70% porosity gyroid design achieved a favorable balance among fluidic permeability, mechanical resilience, osteogenesis, and angiogenesis. To further enhance the intrinsic bioactivity of these architected matrices, Oliveira et al. [90] doped strontium (Sr) into the BCP framework; this elemental substitution improved osteogenic potential while preserving the biomimetic inorganic backbone. Extending this strategy toward therapeutic delivery, Dahiya et al. [91] integrated carvacrol-loaded nanoparticles into 3D-printed SiO_2_/tricalcium phosphate (TCP) scaffolds, allowing the osteoconductive architecture to provide localized antibacterial and anti-inflammatory effects.

Despite these major topological and biological advancements, the inherent lack of fracture toughness in ceramics remains a clinical hurdle. Addressing this structural fragility at the materials level, Zhang et al. [92] improved the biomechanical resilience of printed bioceramics for critical-sized bone defect reconstruction through the induction of in-situ-oriented grain growth, reducing fracture susceptibility through internal crystalline architecture control (Fig. 5B). Along the direction of multifunctional design, Yan et al. [93] functionalized TPMS ceramic scaffolds with a dual-delivery system of bone morphogenetic protein-2 (BMP-2) and zoledronic acid, creating an osteoimmune niche capable of simultaneously promoting osteogenesis while inhibiting premature osteoclastic resorption. Overall, CaP ceramic-based metamaterials have achieved progress in supporting osseointegration. Nevertheless, addressing intrinsic brittleness, securing long-term load-bearing fatigue resistance, and synchronizing the kinetics of ceramic dissolution with the volumetric deposition of new bone remain challenges that remain to be resolved.

#### Bioactive glasses

Compared with traditional CaP ceramics, bioglass-based metamaterials, particularly those integrating MBG, place greater emphasis on controlled ion release kinetics and expansive therapeutic delivery capacity. Demonstrating this multifaceted utility, Zhao et al. [94] strategically incorporated cerium-doped MBG nanoparticles into 3D-printed bioceramic scaffolds as a dynamic reinforcing phase; this integration improved mechanical resilience and local antioxidant capacity and in vitro osteogenic performance. This establishes that the mesoporous phase goes beyond its traditional role as a passive molecular carrier, emerging as a component for both structural reinforcement and active biological regulation. Supporting this potential, García-Lamas et al. [95] showed within a critical-sized rabbit long-bone defect model that architected MBG-enriched scaffolds functioned as bone substitutes and supported in vivo bone regeneration across the defect interface.

Based on this delivery advantage, contemporary bioglass designs have shifted toward disease-specific therapeutic interventions. For instance, Qi et al. [96] engineered MBG platforms to deliver osteoinductive long noncoding RNAs derived from bone marrow stromal cell (BMSC) extracellular vesicles, achieving improved structural repair in compromised senescent bone defects. Furthermore, MBG coatings have been used to amplify the interfacial bioactivity of borosilicate bioactive glass, establishing a basis for hierarchical, multiscale functionalization [97]. Despite these advantages, compositional differences remain important; Pacheco-Vergara et al. [98] demonstrated that 3D-printed MBG, 45S5 bioglass, and β-TCP exhibit clear differences in printability and baseline bioactivity, indicating the need for careful material selection. Extending compositional design, Dai et al. [99] introduced magnesium doping into micro/nanobioglass composite scaffolds, coordinating simultaneous osteogenesis, angiogenesis, and osteoimmune modulation, while de Carvalho et al. [100] integrated CaP and MBG into a unified, printed architecture. Overall, the central advantage of bioglass-based metamaterials resides in their capacity for ion-mediated regulation and therapeutic payload delivery. Nevertheless, establishing processing stability, ensuring long-term mechanical fatigue reliability, and clarifying the in vivo performance variances across distinct glass compositions remain imperative research areas.

#### Functional ceramics and cements

Beyond the canonical domains of CaPs and conventional bioactive glasses, several ceramic systems that are difficult to print or more oriented toward functional devices are gradually being integrated into skeletal reconstruction. Extending traditional clinical materials, Hiepe et al. [101] demonstrated that magnesium phosphate and calcium magnesium phosphate bone cements could be successfully 3D printed into hierarchically porous architectures, transitioning bone cements from amorphous void fillers into structurally designed repair scaffolds. Concurrently, translating these designs into clinically relevant models, Marshall et al. [102] showed in a sheep femoral condyle defect that Laponite–BMP-2-functionalized 3D-printed ceramic scaffolds yielded in vivo consolidation, marking a shift from small-animal exploratory studies toward large-animal validation.

Functional ceramic systems have also been used to integrate biophysical stimulation into scaffold matrices. Wu et al. [103] combined barium titanate with calcium silicate to engineer ceramic scaffolds capable of providing structural support and localized piezoelectric stimulation for large-defect repair. Chen et al. [104] used ZnO-modified ultrasound (US)-responsive piezoelectric ceramics for infected bone defects, extending electroactive ceramic scaffolds to complex repair settings. However, ceramic-rich systems still face brittleness and limited shape adaptability. Hu et al. [105] addressed this limitation through flexible biopolymer-assisted printing, improving the structural compliance and processing stability of ceramic scaffolds. Overall, functional-ceramic- and bone-cement-based metamaterials broaden the compositional repertoire for bioactive scaffold design, but long-term load-bearing integrity, manufacturing consistency, and clinical scalability still require further validation.

### Polymeric metamaterials

Polymeric metamaterials provide conformability, controlled degradation, and high functionalization capacity. Natural and synthetic polymers can form hierarchical pores, directional channels, drug delivery domains, and shape-changing structures [106,107]. Their processing at relatively mild temperatures also facilitates incorporation of cells, proteins, and labile therapeutics. Here, polymer systems are included when architecture directly controls mechanical, transport, or biological behavior rather than when a polymer is used only as a bulk carrier. Their principal limitation is insufficient stand-alone strength and space maintenance in large load-bearing defects, which often requires reinforcement or hybridization.

In natural polymers, current design strategies focus on replicating the architecture of the ECM while reinforcing macroscopic structural stability. For example, Hu et al. [108] engineered a hierarchically porous nanofibrous scaffold that integrated an ECM-mimicking fibrillar network with interconnected macropores, suggesting that the coexistence of multiscale fibers and porosity supports cellular adhesion and deep volumetric migration (Fig. 5C). To circumvent the limited mechanical stability of pure natural polymers, Jovanović et al. [109] utilized genipin-cross-linked gelatin, improving the scaffold’s mechanical resilience without compromising its inherent cytocompatibility. Building on this concept, Zhong et al. [110] developed a composite silk fibroin and hydroxypropyl methylcellulose scaffold that supported bone regeneration through predictable degradation kinetics and good in vivo biocompatibility, while Liu et al. [111] improved uniform cellular distribution and new bone volume utilizing multiscale 3D-printed silk porous hydrogels (Fig. 5A). Beyond monolithic designs, Feng et al. [112] constructed a bilayer periosteum-like scaffold utilizing silk fibroin and sodium alginate, advancing natural polymer scaffolds from single-layer defect fillers into tissue-biomimetic stratified constructs. Nonetheless, natural polymer scaffolds remain constrained by their limited load-bearing capacity, frequently struggling to maintain critical anatomical space in large load-bearing defects without the synergistic integration of synthetic polymers, reinforcing inorganic phases, or active therapeutic delivery systems.

Conversely, synthetic-polymer-based metamaterials offer structural plasticity and are useful for coordinating multistage therapeutic interventions. In polymer/inorganic hybrid hydrogel systems, Yang et al. [113] engineered a xonotlite nanofiber bioactive 3D-printed hydrogel scaffold for vascularized bone regeneration, while Cooke et al. [114] showed that 3D-printed polyurethane architectures could withstand the biomechanical demands of bone regeneration, suggesting that polymer platforms are not limited to soft tissue engineering. Moving beyond isotropic sponge-like architectures, Cheng et al. [115] developed a hierarchical scaffold with directional microchannels; this anisotropic topology accelerated cellular infiltration and vascular ingrowth into the core of thick scaffolds. Jalali et al. [116] incorporated salicin into poly(ε-caprolactone) (PCL) architectures to improve femoral defect repair. Dong et al. [117] embedded a low-polymer-content hydrogel within a rigid 3D-printed poly(lactic-*co*-glycolic acid) (PLGA) framework to achieve sustained local BMP2 release for large segmental defects, whereas Romero-Torrecilla et al. [118] combined PCL with PLGA microparticles to engineer scaffolds with tunable drug release. Shen et al. [119] used dynamic culture to improve nutrient transport and osteogenic maturation within PCL and gelatin methacryloyl (GelMA) scaffolds. Liu et al. [120] embedded biomineral- and vascular endothelial growth factor (VEGF)-functionalized micro/nanofibers into 3D-printed GelMA hydrogels, achieving sustained calcium and phosphate ion release, endothelial activation, and coupled osteogenesis and angiogenesis in critical-sized cranial defects. Zhang et al. [121] linked neurogenesis with bone regeneration using a microsphere hydrogel platform. More recently, scaffolds with multistage osteogenic activity have been reported by Li et al. [122] and Zhou et al. [123], suggesting that polymer-based scaffolds are beginning to move from static implants toward temporally staged therapeutic systems. Overall, polymer-based metamaterials provide plasticity, loading capacity, and advantages for staged intervention, but their standalone use in large load-bearing defects remains limited by insufficient intrinsic stiffness and long-term space maintenance.

### Composite metamaterials

Composite metamaterials represent an integrated strategy for structural and compositional design, shifting bone defect treatment from single-material void filling toward coordinated tissue regeneration. Rather than simply blending materials, these platforms integrate metals, bioceramics, bioglasses, polymers, 2D nanophases, therapeutics, and exosomes according to specific structural and biological tasks. Similar composite design principles have been widely used in energy systems, catalysis, electronic devices, and protective structures [124], while their translation into orthopedic reconstruction allows one scaffold to combine mechanical support, inorganic mineralization, cell recruitment, vascularization, anti-infection activity, and osteoimmune regulation. Early studies mainly explored organic–inorganic hybridization. Wang et al. [125] engineered a magnesium silicate and polylactic acid composite scaffold that coupled neuroregenerative, angiogenic, and osteogenic functions. Emadi et al. [126] showed that incorporating calcium zirconium silicate into PCL architectures enhanced interfacial bioactivity, while Dai et al. [127] induced in situ biomimetic hydroxyapatite mineralization on nanoattapulgite and PCL scaffolds to improve cranial defect repair. To further coordinate degradation with bioactivity, Mirzavandi et al. [128] integrated ordered mesoporous calcium magnesium silicate into PCL and gelatin matrices, achieving a synchronized gradient of degradation and osteogenesis. In this section, composite metamaterials are discussed when phase distribution, pore architecture, or stimuli-responsive organization contributes directly to mechanical, transport, or biological behavior.

As composite fabrication strategies rapidly matured, research shifted toward engineering scaffolds capable of navigating compromised, disease-specific pathological microenvironments. Confronting posttumor reconstruction, Huang et al. [129] constructed a trielement-doped hydroxyapatite (HA) and PCL scaffold capable of driving bone regeneration following osteosarcoma resection (Fig. 6A), while Chen et al. [130] deployed PCL and strontium-doped hydroxyapatite@deferoxamine (SrHA@DFO) architectures to couple osteogenesis with critical neovascularization. Amplifying this cell-instructive capacity, studies by Rocha et al. [131] and Shaikh et al. [132] demonstrated that marine-derived collagen synergized with Sr-doped CaPs and Sr-substituted nanohydroxyapatite, respectively, establishing stable, osteoinductive composite frameworks. Subsequent innovations increasingly emphasized dynamic interfacial reconstruction and functional surface modifications. Du et al. [133] pioneered cell-instructive composite scaffolds utilizing negative mold casting, and Han et al. [134] grafted Ti_3_C_2_ MXene alongside 3,4-dihydroxyphenylalanine–stromal-cell-derived factor 1α onto PCL composites to improve stem cell recruitment and osteogenic differentiation. Confronting the clinical challenge of infected defects, Chen et al. [135] engineered a vancomycin-eluting poly(glycolide-*co*-caprolactone) and MXene bifunctional scaffold, coupling antibacterial activity with skeletal regeneration. Introducing stimuli-responsive “smart” properties, Li et al. [136] developed a thermoplastic polyurethane (TPU) and polyvinylidene difluoride biphasic polymer scaffold characterized by shape memory and intrinsic piezoelectric effects, coupling structural adaptation with localized electrical stimulation to promote tissue repair [136]. Similarly, specialized hybrid systems, including nanohydroxyapatite (nHA)/methacrylated silk fibroin and erythropoietin-loaded silk fibroin, collagen, and hydroxyapatite scaffolds, have broadened the application of composites in complex cranial and alveolar reconstructions [137,138].

**Fig. 6.**
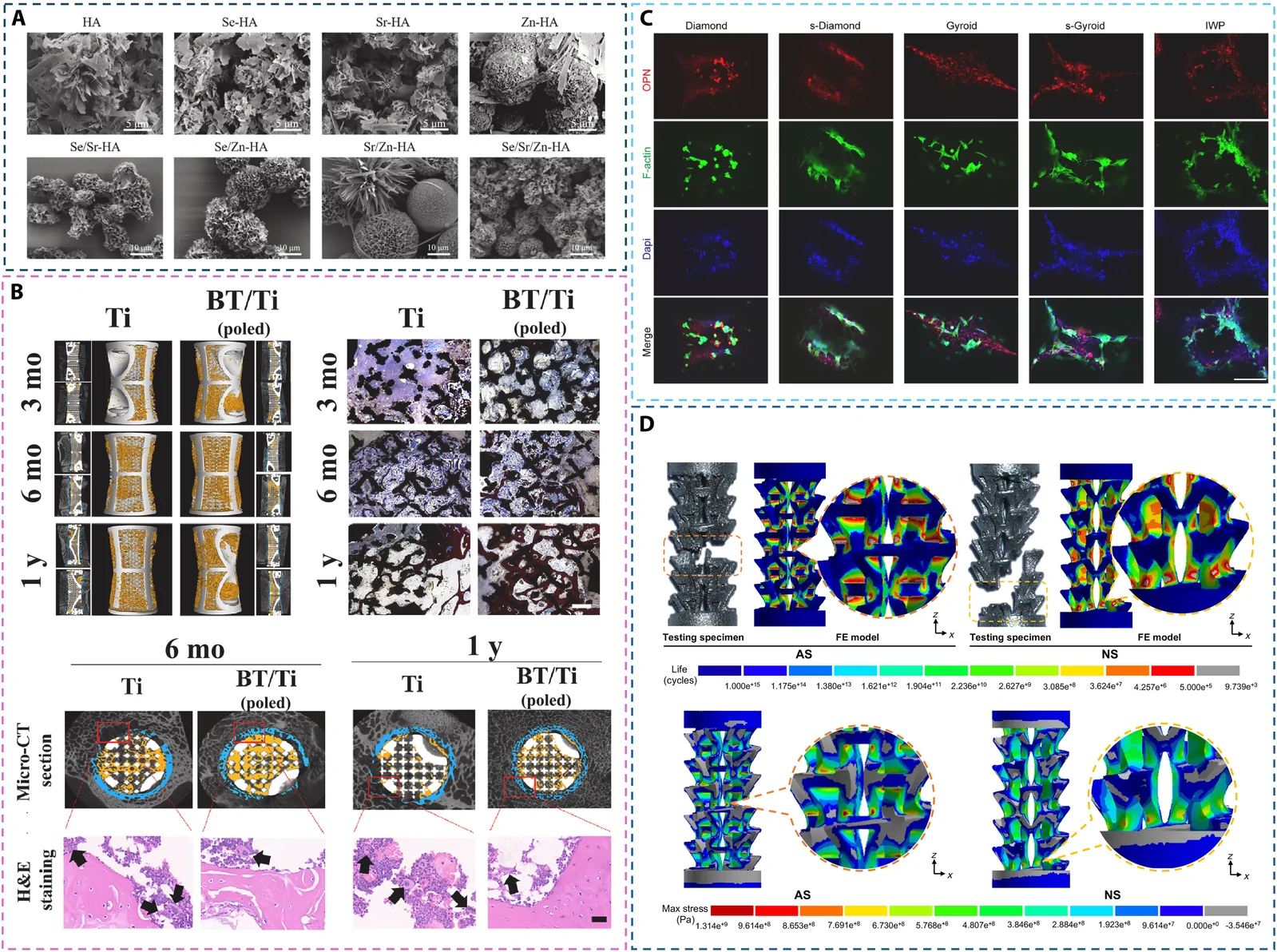
(A) Morphological characterization of Se/Sr/Zn-doped hydroxyapatite components used for multifunctional composite scaffolds [129]. Copyright 2023, Elsevier. (B) Micro-computed tomography (CT) and histological evaluation of piezoelectric BaTiO_3_-coated Ti6Al4V scaffolds for bone repair [192]. Scale bars, 2 mm and 50 μm (H&E staining). Copyright 2022, Elsevier. (C) Confocal imaging of BMSCs cultured on TPMS bioceramic scaffolds with different architectures [166]. Scale bar, 200 μm. Copyright 2023, Elsevier. (D) Finite element (FE) and fatigue evaluation of auxetic and nonauxetic porous bone screw architectures [179]. AS, auxetic porous bone screw; NS, nonauxetic porous bone screw. Copyright 2023, Elsevier. H&E, hematoxylin and eosin.

Overall, composite metamaterials possess the compositional flexibility required to match complex disease-specific microenvironments. Tan et al. [139] designed an alginate, HA, and small intestinal submucosa scaffold for diabetic bone defects, while Kim et al. [140] integrated natural polyphenols into PCL, collagen, and alginate networks to expand the biological regulatory capacity of composite platforms. Local microenvironmental regulation has also been explored through Sr/Mg-containing bioactive glass composites [141] and magnetic MBG nanocomposites designed to enhance osteogenesis and angiogenesis [142]. Janmohammadi et al. [143] showed that PCL/tragacanth gum/bioactive glass scaffolds improved scaffold bioactivity, stiffness, cell viability, and osteogenic differentiation. Further incorporation of cellulose nanocrystals may provide an additional route to improve mechanical and biological performance [144]. Additional composite designs have extended this strategy through different routes. Ceramic-coated PCL scaffolds, GelMA-SrHA hybrid hydrogels, PCL/HA bionic scaffolds, PCL/silk fibroin/β-TCP scaffolds, and hyaluronic acid methacryloyl (HAMA)/α-TCP hydrogels have been used to improve osteoconductive architecture and bone repair [145–149]. Linezolid-loaded PLGA/nHA scaffolds address infected bone defects [150]. GelMA/nHA/melanin, DFO-loaded GelMA-coated poly(glycolide-*co*-caprolactone)–HAPhydroxyapatite, and nHA/polyamide 6,6 porous scaffolds link osteogenesis with immunomodulation, antioxidant regulation, angiogenesis, or vascularized bone regeneration [151–153]. Cell spheroids and sulfonated polyetheretherketone/bioactive glass scaffolds provide additional strategies for improving bone regeneration in specific defect models [154,155]. In complex pathologies, stimuli-responsive composite modules can perform temporally distinct tasks: Black phosphorus photothermal-responsive hydrogels alleviate oxidative stress and remodel bone homeostasis in osteoporotic injuries [156], whereas Sr, silk fibroin, and HA-functionalized scaffolds promote regeneration through immunomodulation and sequential angiogenic–osteogenic coupling [157]. Composite metamaterials are therefore useful for complex defects, but they are also constrained by system complexity, reproducible manufacturing, quality control, and temporal synchronization among functional modules. Overall, no single material platform is universally superior. Metal-based systems are suited for long-term load-bearing support, bioceramic and bioglass platforms for ion-mediated regeneration, polymeric systems for staged delivery and conformability, and composites for severe multipathology defects. Having established these material foundations in the “Material Platform” section, the “Functional Strategies” section discusses the functional strategies through which these architected platforms intervene in the bone regeneration cascade. A practical material hierarchy should begin with the defect rather than the newest functional component. Required load-bearing duration, defect geometry, vascular access, infection status, and expected remodeling rate determine whether a permanent metal, degradable metal, ceramic, polymer, or composite is appropriate. Single-phase systems are preferable when they can satisfy the dominant requirement because they simplify manufacturing and regulation. Composite escalation is justified when functions must be spatially separated, delivered sequentially, or maintained over different time scales. The key comparison is therefore not the number of incorporated components but whether each phase has a defined structural or biological role and whether its contribution can be measured independently. Table S1 summarizes the representative material platforms, architectural features, principal biological roles, limitations, and suitable indications discussed in the “Metallic metamaterials”, “Ceramic and bioglass metamaterials”, “Polymeric metamaterials”, and “Composite metamaterials” sections.

## Functional Strategies

Functional strategies are classified by their dominant physical intervention mode. A stimuli-responsive scaffold is a construct whose behavior changes in response to a specified endogenous or externally applied cue. It is discussed as a metamaterial only when designed architecture contributes to that response [158]. Mechanical, electroactive, magnetic, US-responsive, photoresponsive, and shape memory systems are therefore included when their structural organization contributes directly to a mechanical, transport, or stimulus response. A single scaffold can belong to more than one group, but classification is based on the primary mechanism used to alter the repair environment. This framework separates structural function from base material composition and clarifies which stimulus is delivered, how it is transduced, and which biological process is targeted.

### Mechanical regulation

Mechanical metamaterials are architected platforms designed to achieve tunable effective moduli, deformation modes, and stress transfer behaviors through the rational design of lattices, minimal surfaces, auxetic unit cells, and graded topologies. In bone repair, these architectures are mainly represented by TPMS, auxetic, and gradient designs, which have been used to regulate modulus, deformation, pore transport, and stress transfer [159,160]. From a clinical translation perspective, early applications have mainly targeted load-bearing metallic frameworks. Crovace et al. [161] implanted biomimetic porous Ti6Al4V scaffolds into a large 5-cm segmental tibial defect in a sheep model; the animals achieved immediate postoperative weight bearing, culminating in stable bony bridging and suggesting that sustained biomechanical support is a key requirement for large-defect repair. However, passive macrosupport alone does not explain the regenerative differences observed across different topological architectures. Alonso-Fernández et al. [162] compared bioactive polylactic acid scaffolds with distinct filament deposition topographies and found that a helical architecture promoted greater bone regeneration in critical rabbit femoral condyle defects, likely because of improved pore interconnectivity and spatial openness. To improve interfacial activation, Liu et al. [163] superimposed microarc oxidation-derived nanopores and localized strontium doping onto a porous titanium framework, enabling improved mechanical matching and osteogenic activation at load-bearing interfaces. For device integration, Kang et al. [164] combined locking screw fixation mechanics directly into patient-specific porous titanium implants to improve early biomechanical stability and osseointegration. Similarly, Honda et al. [165] combined strontium-eluting intramedullary nails with porous titanium sleeves for long-segment femoral reconstruction, suggesting that mechanical scaffold design can be integrated with fixation strategies and bioactive modification.

Once the load-bearing capacity of mechanical metamaterials was established, research shifted toward pore geometry, mean curvature continuity, and functionally graded designs. Shen et al. [166] evaluated several TPMS bioceramic architectures and found that continuous zero-mean-curvature surfaces, such as diamond, gyroid, and inhibitor of Wnt production (IWP) structures, promoted early new bone ingrowth, whereas sharp-sheet-like pore channels were less favorable for critical defect bridging (Fig. 6C). A multimodal study combining mechanical testing with in vitro and in vivo evaluation also showed that continuous curved topologies improved compressive resilience, cell adhesion, and in vivo osseointegration [167]. However, increasing porosity in curved structures does not automatically improve biological outcomes. Wang et al. [168] showed that pore size, strut wall thickness, and overall porosity jointly determine compressive performance, cellular behavior, and final repair outcomes in gyroid networks. To reproduce the hierarchical heterogeneity of native bone, Li et al. [169] proposed a Haversian-system-like gradient TPMS scaffold with a radial pore size transition from dense periphery to porous core; this architecture improved shear stress distribution, permeability, and stress shielding. In metallic systems, Xiao et al. [170] combined a functionally graded gyroid Ti6Al4V scaffold with modification and achieved a higher bone volume fraction, with bone volume/total volume reaching 27% at 12 weeks in a large rabbit radial defect. Gradient architectures have also been used in tendon bone interface regeneration, where 3D-printed biomimetic gradient scaffolds help reconstruct multitissue enthesis transitions [171]. This concept has further been adapted into bioceramic platforms to develop multifunctional pore designs that combine reduced effective modulus with improved mass transport capacity [172,173].

Mechanical metamaterial design has increasingly moved from empirical topological screening toward computationally guided parameter optimization and device-level integration. Zhang et al. [174] showed that scaffold permeability and effective diffusivity regulate the kinetics of volumetric bone ingrowth, suggesting that static compressive modulus alone is insufficient for evaluating scaffold efficacy. Cancellous-bone-inspired computational fluid dynamics modeling further showed that porosity and localized surface curvature influence wall shear stress distribution, providing a basis for incorporating dynamic fluid microenvironments into structural lattices [175]. Pires et al. [176] used optimization algorithms to explore the TPMS parameter space and balance yield strength, mass transport, and additive manufacturing constraints, while Liu et al. [177] modified scaffold curvature to engineer a pro-osteogenic interfacial niche. This parameter-driven logic has also been introduced into devices; for example, metallic bone screws with internal TPMS-based porous topology have been shown to enhance peri-screw osseointegration and pull-out strength [178]. These device-level studies indicate that structural integrity should be considered together with biological fixation during implant design (Fig. 6D) [179]. In contrast to continuous surface topologies, auxetic metamaterials use negative Poisson’s ratio deformation to redistribute local strain. Auxetic meta-biomaterials fabricated by selective laser melting can improve strength while tuning the effective modulus toward the range relevant to trabecular bone scaffolds [180]. However, static mechanical advantages do not necessarily predict in vivo longevity, and fatigue behavior under physiological cyclic loading remains an important engineering challenge [181]. Yarali et al. [182] further described auxeticity as a mechanobiological tool because anomalous lateral contraction under compression can alter the local strain environment sensed by cells. This concept is supported by distinct adhesion and osteogenic differentiation responses observed in auxetic versus conventional microarchitectures [183]. The mechanical performance of trabecular-mimetic auxetic scaffolds further suggests potential for device-level applications [184]. Currently, comparative studies spanning TPMS versus Voronoi structures, bone-inspired interpenetrating networks, graded-porosity 3D-printed scaffolds, and digital-light-processing (DLP)-fabricated HA scaffolds continue to expand the design space of mechanical metamaterials [185–188]. Some recent designs also suggest that pore architecture may influence the immune microenvironment and macrophage polarization [188]. Overall, mechanical metamaterials have moved beyond simple stress-shielding reduction toward designed platforms that encode load-bearing, mass transport, mechanotransduction, and selected osteoimmune cues. Nevertheless, no universal topology is suitable for all anatomical sites, and increasing topological complexity also increases the need to control additive manufacturing deviations and long-term fatigue failure. For design selection, modulus should be interpreted together with yield strength, permeability, local strain, and fatigue life. Two architectures with similar bulk stiffness can generate different pore wall stresses and fluid shear fields, while small printing deviations can change both mechanical reliability and cellular access. Future comparisons should report as-manufactured geometry, cyclic loading conditions, failure location, and transport metrics alongside biological outcomes. This would allow topologies to be selected for defined anatomical tasks rather than ranked by a single mechanical or osteogenic endpoint.

### Electroactive and magnetic stimulation

Electroactive and magnetically responsive metamaterials for skeletal reconstruction go beyond passive structural support by regulating local electric-field- and magnetic-field-mediated biological processes through conductive networks, piezoelectric domains, magnetically responsive nanoparticles, and composite unit cells. In bone repair, these systems have mainly been developed from electroactive biomaterials and magnetoelectric composite strategies [189,190]. Their significance is twofold. First, they provide local bioelectric and biomagnetic cues that are largely absent in conventional inert implants. Second, when coupled with external or endogenous fields, they can transform an implant from a passive filler into a bioactive interface for sustained local regulation. Among electroactive platforms, piezoelectric and conductive systems were introduced early in this transition. Liu et al. [191] coated piezoelectric BaTiO_3_ nanoparticles onto 3D-printed porous Ti6Al4V architectures, generating a composite scaffold that combined structural support with an electrically active local microenvironment. Wu et al. [192] further showed that polarized BaTiO_3_/Ti6Al4V scaffolds promoted macrophage transition toward an anti-inflammatory phenotype and supported osteogenesis by inhibiting mitogen-activated protein kinase (MAPK)/c-Jun N-terminal kinase signaling and up-regulating oxidative phosphorylation (Fig. 6B). Conductive polymer scaffolds combined with direct current stimulation also enhanced bone regeneration, while HA/TCP composites exposed to electrical fields regulated Wnt-related osteogenic pathways [193,194]. Nanoconductive osteogenic hydrogels promoted Ca^2+^ influx and activated TGFβ/Smad2 signaling to support mineralization, whereas MXene-based conductive hydrogels improved mechanical resilience while supporting neurogenesis and osteogenesis [195,196]. In infected bone defects, conductive hydrogels may help coordinate infection control, osteoimmune regulation, and vascularized bone regeneration [197].

As the field has advanced, electroactive metamaterials have moved beyond externally applied currents toward endogenous piezoelectricity and self-powered or mechanically triggered electrical signals. A periosteum- and bone-inspired hierarchical scaffold harnessed endogenous piezoelectric effects triggered by physiological micromotion to support neurovascularized bone regeneration, suggesting that bioelectrical cues are closely linked to vascular and neural reconstruction [198]. A BaTiO_3_ and polylactic acid biomimetic scaffold further combined shape memory behavior with bioelectrical activity, allowing the implant to adapt to complex defects while providing local piezoelectric stimulation [199]. Self-enhanced piezoelectric scaffolds have also been shown to regulate inflammatory responses, ECM remodeling, and subchondral bone repair through localized electromechanical output, highlighting a link between piezoelectric signals and osteoimmune transition [200]. In parallel, magnetically responsive metamaterials have emerged as another important branch. Their main advantage is that external magnetic fields can regulate the local microenvironment without direct electrical contact or percutaneous wiring. Thus, electroactive and magnetically responsive metamaterials have expanded from local electrical stimulation toward noninvasive magnetic regulation.

Within magnetically responsive systems, the core objective is to translate external magnetic forces into usable biological cues. Maglio et al. [201] implanted porous hydroxyapatite scaffolds doped with Fe_3_O_4_ magnetic nanoparticles into critical sheep bone defects. By applying permanent magnets, they generated a VEGF nanoparticle concentration gradient within the scaffold pores and achieved magnetic-field-guided bone regeneration. This finding suggests that the function of magnetic scaffolds depends not only on the presence of magnetic components but also on their ability to convert external magnetic fields into localized biochemical gradients. Sun et al. [202] further showed that magnetic HA scaffolds regulated macrophage polarization timing through peroxisome-proliferator-activated receptor/Janus kinase–signal transducers and activators of transcription signaling and fatty acid metabolism, shifting the inflammatory microenvironment toward osteogenesis. Magnetic scaffolds fabricated from recombinant structural proteins enhanced osteogenesis by regulating intracellular calcium homeostasis through magnetomechanical transduction [203]. Recent designs have also targeted more complex microenvironments. Reactive-oxygen-species-scavenging magnetic scaffolds under a mild 30-mT static magnetic field up-regulated ALP, RUNX2, and BMP2 while providing antibacterial and antioxidant effects [204]. Static magnetic fields combined with human demineralized bone matrix have also been reported to enhance cranial bone remodeling [205]. Beyond porous architectures, self-powered piezoelectric chips showed that sustained electromechanical stimulation could participate in critical-sized bone defect repair even without a conventional volumetric scaffold [206]. 3D-printed magnetic scaffolds can convert magnetic cues into local osteogenic and angiogenic responses. Recent proteomic evidence further suggests that crystallin αB/phosphatidylinositol 3-kinase (PI3K)–AKT and nuclear factor κB (NF-κB)/HIF-1α/VEGF-related pathways may participate in magnetic scaffold-mediated bone and vessel regeneration [207]. Overall, electroactive and magnetically responsive metamaterials may coordinate immune, metabolic, vascular, and osteogenic responses. However, field dosage windows, stimulation uniformity in deep tissue, and long-term in vivo biosafety still need further clarification. A major methodological issue is that the delivered stimulus is often less well characterized than the scaffold itself. Electrical potential, piezoelectric charge, magnetic flux density, field gradient, exposure duration, and spatial uniformity should be measured under physiologically relevant loading and tissue depths. Without these data, nominally similar systems cannot be compared and biological effects cannot be separated from heating, ion release, or material chemistry. Dose–response studies and sham-field controls are therefore essential for establishing causality and safe operating windows.

### US activation

Compared with mechanical metamaterials, which mainly rely on static structural cues after implantation, US-responsive metamaterials enable repeated activation of the repair niche. These systems use US to trigger localized mechanical, electrical, or therapeutic release responses within defect sites. They were originally developed for wave manipulation and acoustic control and have since been adapted for noninvasive, remotely triggered biomedical therapy. In skeletal reconstruction, their advantage lies in repeated and controllable stimulation of deep defects without secondary surgery [208–210]. Low-intensity pulsed US (LIPUS) combined with scaffolds remains the most common strategy. Volarić et al. [211] showed that LIPUS promoted bone regeneration and up-regulated Osterix and cyclooxygenase-2 in a critical-sized rat calvarial defect model. When combined with porous titanium alloy scaffolds, LIPUS promoted tissue ingrowth and matrix maturation while reducing early osteoclast-mediated resorption [212]. LIPUS with lipid microbubbles enhanced PLGA/TCP scaffold repair, and US-responsive injectable alginate systems enabled on-demand drug release [213,214].

Despite these successes, pure acoustic stimulation relies on the passive mechanotransductive response of host tissues to incident acoustic energy. To go beyond this limitation, sono-piezoelectric metamaterials have been engineered to harvest noninvasive acoustic waves and transduce them into localized bioelectrical signals. Zhu et al. [215] engineered a US-driven piezoelectric guided bone regeneration membrane; under acoustic irradiation, this membrane generated stable piezoelectric output, promoted osteogenesis in rat cranial defects, and achieved a 40% antibacterial effect against localized pathogens. US-enhanced piezoelectric scaffolds were also combined with cell-stretch-induced mechanotransduction for nonload-bearing defect repair, showing that acoustic waves can cooperate with local electrical cues to enhance osteogenic responses [216]. Mechanistic studies further showed that US-activated piezoelectric platforms induced voltage-gated calcium-channel-dependent Ca^2+^ influx in osteoblasts while suppressing NF-κB inflammatory signaling in macrophages, thereby coordinating osteoimmune regulation with osteogenesis [217].

Based on these studies, US-responsive strategies have expanded from simple physical stimulation toward controlled biological regulation. Programmed parathyroid hormone pulsatility coupled with US-activated piezoelectric stimulation has extended this strategy to deep bone defect regeneration [218]. US-responsive piezoelectric membranes have also been applied to osteoporotic bone defect repair, while related studies targeting the EphrinB2/EphB4 axis further explored US-associated regulation of osteoblast–osteoclast communication [219,220]. Li et al. [221] used LIPUS to activate BaTiO_3_-modified sulfonated polyetheretherketone scaffolds, generating stable piezoelectric output and promoting osteogenic differentiation through Piezo1-mediated Ca^2+^ influx and Akt/glycogen synthase kinase 3β (GSK3β)/β-catenin signaling. Overall, US-responsive metamaterials provide a useful noninvasive route for remote and repeated activation of bone repair scaffolds. Before broad translation, acoustic dosage windows, tissue attenuation, and long-term biosafety need to be standardized. Acoustic dose should likewise be treated as a system property rather than a device setting alone. Frequency, intensity, duty cycle, coupling medium, defect depth, and scaffold acoustic impedance jointly determine the local mechanical and electrical output. Standardized mapping of pressure fields and piezoelectric voltage within tissue-mimicking models would improve comparison across platforms. Repeated treatment protocols should also assess whether efficacy is maintained as new tissue fills the pores and changes acoustic transmission.

### Photoresponsive therapy

Photoresponsive metamaterials are stimuli-responsive architectures that use photodynamic or photothermal effects to couple lesion treatment with tissue reconstruction. Reviews have shown that photothermal composite scaffolds have moved from tumor ablation tools toward platforms that also support new bone regeneration [222]. Early in vivo studies further demonstrated that photodynamically active biomaterials could improve histological bone regeneration and long-term osseointegration in periodontal and bone surface defects [223,224]. However, photodynamic or photothermal treatment alone may focus mainly on lesion ablation and may not provide a sufficient bioactive foundation for subsequent volumetric bone repair. Here, photoresponsive systems are discussed as metamaterial-inspired platforms when optical responsiveness is integrated with designed scaffold architecture.

To address this limitation, researchers have developed theranostic photoresponsive platforms. For example, an 5-carboxy-8-hydroxyquinoline (IOX1)-enhanced, 3D-printed silicene scaffold integrated second near-infrared window (NIR-II) photothermal tumor ablation with bone regeneration, suppressing osteosarcoma recurrence while preserving an interfacial microenvironment favorable for osteogenesis [225]. Biomimetic bone periosteum scaffolds further combined photothermal antitumor therapy, large-defect bridging, and spatiotemporal regulation of innervation and ossification networks within one platform. This suggests that light-responsive architectures may serve as platforms for synchronous reconstruction after tumor resection [226]. Dopamine-functionalized calcium-deficient hydroxyapatite scaffolds, NIR-responsive polymeric matrices, and NIR-activated antibacterial hydrogels further linked local antibacterial regulation, angiogenesis, and osteogenesis through different design routes [227–229]. In more severe clinical settings, metal coordination self-assembled scaffolds, NIR-activated protein nanomicelles, and composite nanofibrous matrices have extended photoresponsive systems to postosteosarcoma reconstruction, oral maxillofacial defects, and infected skeletal nonunions, respectively [230–232].

Photoresponsive metamaterial design has therefore emerged at the interface of photothermal repair and localized antitumor therapy. NIR-responsive shape memory scaffolds have been used for shape-adaptive filling and bone regeneration in irregular defects after tumor surgery [233]. The value of these systems is not limited to antibacterial or antitumor therapy; emerging evidence suggests that they may also regulate local metabolic microenvironments and support bone regeneration under abnormal physiological conditions. Overall, photoresponsive metamaterials offer a useful noninvasive route for coordinating local therapy with regeneration. However, limited optical penetration depth, a narrow photothermal safety window, and heterogeneous optical attenuation in deep cortical tissue remain barriers to translation. Translation will require optical dosimetry that links incident power to temperature and reactive species distribution within the full defect volume. Surface temperature alone may underestimate deep hot spots or fail to detect undertreated regions. Real-time thermal mapping, histological assessment of collateral injury, and explicit separation of tumor control and regeneration phases are needed to define clinically safe protocols.

### Shape adaptation

Unlike US-responsive metamaterials, which rely on remote mechanical energy transduction, thermoresponsive and shape memory metamaterials use programmed thermal transitions to alter shape or function after implantation. Some systems can be compressed for minimally invasive delivery and then recover their predefined geometry after thermal triggering. This shape recovery can improve defect fitting, mechanical interlocking, and local physical contact with host tissue [234]. Here, thermoresponsive and shape memory systems are discussed as architected platforms when designed shape change or thermal responsiveness is coupled to scaffold structure.

As a related photothermal-responsive example, Pt(IV) prodrug-releasing scaffolds have been engineered to integrate localized photothermal therapy with sustained bone repair for postosteosarcoma reconstruction [235]. Shape memory polyester scaffolds have also shown that thermally triggered recovery of a designed architecture can improve mechanical stability and osteogenic capacity [236]. Through compositional hybridization, shape memory polymer and bioglass composite scaffolds have been designed to adapt to irregular defect geometries while supporting ion-mediated osteogenic activity. Subsequent in vivo evaluations showed the sustained biocompatibility, space maintenance, and improved regenerative performance of these thermoresponsive networks [237,238].

Extending spatial and temporal control, 4D-printed shape memory scaffolds introduce a time-dependent dimension into skeletal reconstruction and show potential in staged treatment of infected bone defects [239]. Multistep shape memory composite scaffolds and NIR dual-thermal-responsive architectures have further explored antibacterial repair, bone regeneration, and bone-tumor-related treatment scenarios [240]. Overall, thermoresponsive and shape memory metamaterials are useful for adapting to complex defect morphologies and enabling functional regulation after implantation. Nevertheless, translation requires careful control of thermal activations, avoidance of hyperthermia-induced tissue damage, predictable in situ deformation, and validation of long-term thermomechanical stability. Shape recovery should be evaluated by more than the percentage of recovered geometry. Recovery force, fixation strength, activation time, trigger temperature, repeated cycle stability, and pressure exerted on surrounding tissue determine whether adaptation improves integration or causes injury. These parameters should be measured in wet, constrained, and anatomically realistic environments because unconstrained bench-top recovery can overestimate clinical performance.

## Regenerative Mechanisms

The regenerative efficacy of metamaterials extends beyond the passive osteoinductive properties of their constituent base materials. These engineered architectures shape the local defect microenvironment through structural cues and stimuli-responsive behavior. In irregular bone defects, Mao et al. [241] engineered a water-responsive silk fibroin and magnesium composite scaffold that achieved anatomical conformance through surgical trimming and water-triggered shape recovery while maintaining structural stability, angiogenesis, and biomineralization during degradation. Lu et al. [242] developed an instant-fixation, self-adaptive scaffold to improve interfacial fitting, recruit endogenous skeletal stem cells, and support local angiogenesis in cranial defects. Xuan et al. [243] showed that an ordered bredigite scaffold regulated macrophage polarization and promoted bone regeneration in an onlay graft model, suggesting that ordered architecture can act as a structural cue for osteoimmune regulation. These studies provide representative examples of how structural adaptation, fixation, and ordered architecture can influence local repair responses. More broadly, pore interconnectivity, micro- and nanoscale surface topography, mean curvature distribution, and dynamic mechanical loading can influence cell adhesion, cytoskeletal tension, and mass exchange. These changes are often linked to osteogenic signaling pathways such as YAP/transcriptional coactivator with PDZ-binding motif (TAZ), integrin/focal adhesion kinase (FAK), Wnt/β-catenin, and BMP/Smad in subsequent mechanistic studies [244]. Therefore, this section focuses on how the structural geometries and stimuli-responsive strategies discussed above are translated at the cellular and molecular interface.

### YAP/TAZ mechanotransduction

YAP/TAZ serve as representative transcriptional coactivators of the Hippo signaling pathway. They regulate downstream gene expression through interactions with transcriptional enhanced associate domain family transcription factors [245] and participate in cell proliferation, apoptosis, and lineage commitment [246]. Their nuclear activity is regulated not only by the Hippo–large tumor suppressor kinase cascade but also by biophysical cues from the extracellular environment. In skeletal tissues, YAP/TAZ nucleocytoplasmic shuttling is associated with periosteal osteoprogenitor expansion, skeletal development, and fracture repair. Therefore, when the topological boundaries, strut curvature, and surface mechanics of metamaterials interact with host tissue, YAP/TAZ may act as early nuclear relays that translate mechanical cues into cellular responses [247,248].

In skeletal tissues, YAP subcellular localization contributes to bone homeostasis, while related mechanotransductive studies link YAP/TAZ activity to osteogenic differentiation and bone repair responses [249–253]. Matrix stiffness, pore curvature, and BMP-2-related mechanotransduction can converge on the YAP/TAZ axis [250,252,253]. Surface morphology may also regulate osteogenic differentiation through epigenetic and mechanobiological mechanisms [251]. Metamaterials use these principles by modulating multiscale topology and the local mechanical microenvironment. Modaresifar et al. [254] showed that high-aspect-ratio nanostructured biomaterials increased cell adhesion, promoted YAP nuclear translocation, and converted local mechanical stress into osteogenic signals such as Runx2. Wei et al. [255] also observed YAP/TAZ nuclear accumulation on microroughened titanium surfaces; silencing YAP/TAZ reduced osteogenic markers including Runx2 and bone sialoprotein. Tong et al. [256] reported that YAP up-regulation depends partly on F-actin rearrangement and cooperation with megakaryoblastic leukemia 1 during osteogenic commitment. Kong et al. [257] further showed that TiO_2_ nanotube arrays promoted BMSC osteogenic differentiation in vitro and YAP-related bone formation in vivo, with Piezo1 acting as a downstream mechanosensitive channel. Pan et al. [258] also showed that YAP promotes osteogenesis, suppresses adipogenic differentiation, and helps maintain bone homeostasis in osteolineage cells.

Because scaffold architecture is not sensed directly, nanotopography, adhesion geometry, and local deformation must first be transduced at the cell membrane [259,260]. Fluid shear or matrix rigidity can open Piezo1/2, leading to Ca^2+^ influx and calcineurin-dependent activation of nuclear factor of activated T cells, YAP1, and β-catenin in osteogenic cells [261]. This relationship places Piezo1/2 upstream of a transcriptional network whose magnitude and duration remain dependent on the cell type and material. Time-resolved localization, genetic perturbation, and local mechanical measurements are therefore needed to distinguish pathway dependence from a general association.

### Integrin/FAK signaling

Compared with nuclear mechanotranscriptional relays such as YAP/TAZ, integrin/FAK activation occurs during the early stage of metamaterial-mediated bone repair and serves as a key step in cellular interface recognition [262]. Integrins are heterodimeric transmembrane adhesion receptors that connect the ECM to the cytoskeleton and mediate bidirectional transmission of mechanical and biochemical signals [263]. Although integrin adhesion complexes are involved in tumor cell migration, invasion, and metastasis [264], they are also central to skeletal physiology, where osteocyte integrins regulate load sensing and anabolic responses through PI3K/AKT signaling [265]. When integrins bind ECM ligands and cluster at the cell–material interface, they recruit and activate FAK, initiating the classical integrin–focal adhesion signaling cascade [266]. FAK then undergoes autophosphorylation at Y397 and interacts with Src to amplify downstream MAPK/extracellular-signal-regulated kinase and PI3K/AKT signaling [267]. Beyond tissue repair, FAK signaling is also relevant to tumor invasion and anticancer therapeutic strategies [268,269]. Thus, the integrin/FAK axis is both an entry point for material recognition and an early mechanosensory node that translates structural cues into intracellular responses. Qin et al. [270] further showed that osteocyte β3 integrin is important for bone mass accumulation and load-induced osteogenesis. Together, these findings indicate that integrin/FAK signaling is relevant to bone mechanotransduction and load-induced osteogenesis [265,270], while FAK also represents an important target in tumor biology and anticancer therapeutic strategies [268,269].

When cells first contact a metamaterial scaffold, the material surface first undergoes protein adsorption, followed by cell spreading, pseudopod extension, and focal adhesion maturation [271,272]. These events influence the efficiency of later osteogenic responses. Kang et al. [273] showed that ordered 90-nm TiO_2_ nanotube arrays inhibited osteoclastogenesis and enhanced osteogenic activity through integrin αvβ_3 _activation, indicating that integrin-mediated effects are not limited to osteoblasts. Jinsheng et al. [274] found that TiO_2_ nanotube-based micro/nanotopography activated Thy-1- and integrin/FAK-related pathways and promoted osteogenic differentiation through an F-actin/YAP relay. Liu et al. [275] further showed that 3D-printed titanium scaffolds with hierarchical micro/nanotopography increased osteocalcin (OCN) and CD31 expression through integrin-mediated mechanotransduction, thereby coordinating osteogenic–angiogenic coupling. Long et al. [276] used single-cell RNA sequencing to evaluate the response to scaffold-architecture-induced stress stimulation and found enhanced BMSC proliferation and osteogenic commitment, together with changes in the balance between osteoclastic resorption and osteoblastic deposition.

The preceding evidence places integrin-dependent focal adhesions at the interface between scaffold geometry and intracellular signaling. Adsorbed ECM proteins and pore wall geometry influence integrin clustering, focal adhesion formation, and FAK/Src signaling. The resulting actin organization can influence YAP/TAZ. In osteoblasts, fluid shear also engages a FAK/Src route to PI3K/AKT and β-catenin [277]. Surface chemistry, cell identity, and loading history determine whether FAK signals through actin, PI3K/AKT, MAPK, or more than one route. Studies should therefore test defined adhesion complexes across scaffold surfaces and poorly perfused cores in load-bearing models rather than equating FAK phosphorylation with uniform osseointegration.

### Wnt/β-catenin signaling

The canonical Wnt/β-catenin signaling axis, comprising Wnt ligands, Frizzled receptors, LRP5/6 co-receptors, and the cytoplasmic β-catenin destruction complex, is a central regulator of bone formation, skeletal homeostasis, and bone-related disease [278,279]. Under resting homeostatic conditions, the destruction complex (constituted by Axin, adenomatous polyposis coli, GSK3β, and casein kinase 1) continuously phosphorylates β-catenin, designating it for rapid proteasomal degradation. However, upon the binding of activating ligands (e.g., Wnt3a and Wnt10b) to the Frizzled/LRP5/6 receptor complex, destruction complex activity is inhibited. This allows β-catenin to accumulate in the cytoplasm and subsequently translocate into the nucleus, where it complexes with T cell factor/lymphoid enhancer factor (TCF/LEF) transcription factors to coordinate the expression of an osteogenesis-related gene network. In the context of metamaterial-mediated bone repair, this Wnt/β-catenin cascade influences ECM secretion and mineralization by mature osteoblasts. Abuna et al. [280] demonstrated that modifying the nanotopography of titanium surfaces promoted Wnt/β-catenin signaling and osteoblast differentiation; targeted interference with Frizzled-4 reduced this structural osteogenic effect. Li et al. [281] further showed that nanotopography does not directly shuttle β-catenin into the nucleus; rather, it first modulates cytoplasmic YAP through autophagy-related processes, which subsequently regulate β-catenin nuclear translocation, suggesting a sequential mechanobiological relay between YAP and Wnt pathways. Yu et al. [282] found that micro- and nanohierarchical titanium topographies promoted osteogenic differentiation compared with simple microstructured or smooth surfaces, accompanied by rho-associated protein kinase activation, increased Wnt5a expression, and β-catenin accumulation. Pharmacological inhibition of rho-associated protein kinase reduced this Wnt5a-related osteogenic effect.

These studies have linked specific geometrical parameters to Wnt/β-catenin activation. In a study of biomimetic wrinkled topographies, Zhang et al. [283] showed that osteogenic activation is not a universal response to all patterned surfaces. A surface with an amplitude of 0.7 μm and a wavelength of 3 μm was more favorable for ALP, OCN, osteopontin (OPN), and collagen type I (COL-1) expression, whereas surfaces with larger dimensions showed inhibitory effects. This difference was associated with autophagy-mediated Wnt/β-catenin activation. Zhang et al. [284] also showed that nanoporous titanium surfaces accelerated osteogenesis through the Piezo1/acetyl-coenzyme A/β-catenin cascade. In this process, nanotopography first activated Piezo1 and triggered intracellular calcium influx. This influx increased mitochondrial oxidative metabolism and cytoplasmic acetyl-coenzyme A levels, which then enhanced β-catenin acetylation stability and osteogenic transcription. Together, these studies suggest that Wnt/β-catenin signaling integrates several upstream-material-induced changes, including surface topology, autophagy, mechanosensitive ion channel activation, metabolic regulation, and cytoskeletal remodeling into osteogenic transcription and mineralization.

Because the preceding evidence places Wnt signaling downstream of several material-dependent inputs, its relationship with BMP signaling needs separate consideration. Wnt ligands signal through Frizzled and LRP5/6 to inhibit the Axin/adenomatous polyposis coli (APC)/GSK3β destruction complex, allowing β-catenin and TCF/LEF activity. In osteoblasts, β-catenin and TCF can stimulate BMP2 transcription [285], whereas BMP-2 can increase Lrp5 and reduce β-transducin repeat-containing protein, which stabilizes β-catenin [286]. Pathway reporters and spatially resolved measurements across healing stages are needed before a common causal sequence is assigned to different metamaterial platforms.

### HIF-1α/VEGF signaling

Effective skeletal regeneration is not determined only by increased osteogenic markers. The survival, consolidation, and maturation of new bone within large defects depend on sustained microvascular perfusion, oxygen supply, and nutrient exchange. HIF-1α is a key transcriptional regulator of the hypoxic response. After stabilization under low-oxygen tension, HIF-1α translocates into the nucleus and induces angiogenesis-related molecules, especially VEGF. VEGF then regulates endothelial cell migration, capillary sprouting, and the stabilization of newly formed vessels [287]. In metamaterial-mediated bone repair, HIF-1α/VEGF signaling represents one important route through which local hypoxic cues are translated into vascular reconstruction, helping determine whether a poorly perfused defect region can develop into vascularized bone tissue.

Building on this concept, contemporary metamaterial research has shifted from simple porous metallic supports toward coordinated osteogenic–angiogenic design. Qin et al. [83] proposed an innovative 2-stage deformation metamaterial that moved beyond traditional bulk modulus matching. Despite having a low effective modulus (13 MPa), the scaffold retained load-bearing strength. In a rabbit critical ulnar defect model, this compliance produced a macroscopic callus strain exceeding 2% and was accompanied by up-regulation of calcium ion channels and HIF-1α signaling. At 4 weeks, the new bone fraction was 44% and 498% higher than that of conventional 500- and 13-MPa classical architectures, respectively. This suggests that designed deformation modes can influence vascularization and osteogenesis without relying only on exogenous biologics. Gao et al. [288] further engineered porous Ti6Al4V scaffolds with magnesium-functionalized coatings. This interface enhanced MC3T3-E1 proliferation, ECM mineralization, and ALP activity, while also promoting human umbilical cord endothelial cell adhesion, migration, and capillary tube formation, accompanied by increased HIF-1α/VEGF expression.

The vascular response then depends on whether hypoxic signaling produces functional perfusion within the scaffold. Under hypoxia, stabilized HIF-1α increases VEGF production. Osteoblast-specific HIF-1α activation increased vascularity and bone repair in a VEGF-receptor-dependent manner [289]. VEGF-A binds endothelial VEGFR2 and activates PI3K/AKT and extracellular-signal-regulated kinase/MAPK, which support endothelial proliferation and sprouting. In a 3D-printed porous titanium scaffold, hierarchical micro/nanotopography and graded release of silicon ions and VEGF increased BMSC osteogenesis, human umbilical cord endothelial cell angiogenesis, and in situ vascularized bone regeneration [290]. In a rabbit femoral defect model, sequential release of VEGF and BMP-2 from core–shell microspheres in a 3D-printed titanium lattice supported staged angiogenic and osteogenic coupling [291]. HIF-1α overexpression and deferoxamine release provide 2 additional routes for creating a provascular microenvironment [292,293]. These examples show why vessel penetration, lumen formation, perfusion, stability, and coupling to mature bone in the scaffold core should be evaluated rather than early endothelial marker expression alone.

Taken together, these interactions provide a mechanistic framework linking scaffold architecture and surface cues with osteogenic and angiogenic responses. The relationships among mechanosensing, osteogenic signaling, and vascular regulation are summarized in in Fig. S1.

## Translation and Outlook

Metamaterials extend bone scaffolds beyond passive defect filling by coordinating chemistry, architecture, and physical responsiveness. Metallic, ceramic, polymeric, and composite systems can provide load transfer, tissue ingrowth, vascularization, osteoimmune regulation, infection control, and posttumor reconstruction. Their value lies in organizing these functions at the architectural level and matching them to the spatial and temporal demands of healing. US-activated piezoelectric scaffolds illustrate this integration by coupling calcium influx in osteoblasts with reduced NF-κB signaling in macrophages and increased BMP-2 and VEGF secretion [217]. However, combining multiple effects does not automatically improve translation. The key question is which functions are necessary, measurable, and manufacturable for a defined clinical indication. A Ti–polydopamine–Zn coating combined antibacterial activity with enhanced osseointegration under infected conditions, illustrating a simple strategy for multifunctional implant interfaces [294].

Four barriers remain. First, relationships among topology, composition, and biological outcome are still derived largely from empirical screening. The scaffold-architecture-induced stress stimulation framework links topology-dependent mechanics with focal adhesion signaling and shifts in osteoblast–osteoclast balance [276]. Second, mechanistic evidence for YAP/TAZ, integrin/FAK, Wnt/β-catenin, HIF-1α/VEGF, and BMP/Smad signaling often relies on simplified cultures, pharmacological inhibitors, or single in vivo time points. The order and duration of pathway activation across immune, vascular, and skeletal cell populations remain poorly resolved. Third, long-term fatigue, synchronized degradation, deep-core vascular maturation, functional recovery, and revision feasibility remain underreported. Fourth, added biologics, nanoparticles, and electromechanical components complicate manufacturing, sterilization, storage, cost control, and regulatory standardization. These constraints should be treated as design inputs rather than problems deferred until late development. Excessive complexity can become a translational liability even when short-term biological outcomes improve.

### Translational requirements for complex systems

The barriers outlined above show that each added function needs a defined clinical purpose. An added coating, carrier, or active component should be compared with the same architecture and base material without that component. Its inclusion is justified only if it retains function after manufacturing, sterilization, and storage and improves an end point relevant to the intended defect. The comparison should also report the added fabrication steps, quality control requirements, and cost. For permanent systems, the essential evidence concerns cyclic fatigue, fixation, imaging, and the feasibility of revision. For degradable systems, the key question is whether loss of strength, degradation products, host response, and new bone formation remain coordinated over time.

The model should then match the intended device use. Rodent studies are suitable for mechanism and dose screening. Rabbit models accommodate larger defects and more demanding geometry, but they do not reproduce the loading or remodeling conditions of human segmental reconstruction. When a device is intended for weight-bearing or segmental reconstruction, sheep or canine studies should test fixation, cyclic loading, vascularized bone formation, and functional recovery. Across these models, studies should report manufacturing deviation, physiologic fatigue after implantation, degradation trajectory, deep-core perfusion, revision feasibility, and long-term outcome [64,102].

Accordingly, future work should start from the clinical indication rather than add functions without a defined purpose. Customized 3D-printed titanium grids have already supported mandibular reconstruction in large animals and 3 patients, with bone ingrowth and no reported mechanical complications [64]. This experience illustrates the value of clear anatomical indications, controllable manufacturing, and device-level fixation. Load-bearing defects require durable mechanics and stable osseointegration, whereas infected, osteoporotic, diabetic, and posttumor defects require disease-matched control of infection, immunity, and vascularization. Preclinical evaluation should extend beyond early osteogenic markers to long-term remodeling, neurovascular ingrowth, interface stability, fatigue, degradation, and functional recovery. Standardized reporting of geometry, manufacturing deviation, loading conditions, and degradation would also improve cross-study comparison. The most promising systems will combine a clear indication, scalable manufacturing, controllable risk, and clinically relevant evidence.

## Data Availability

The data that support the findings of this study are available from the corresponding author upon reasonable request.
