# Supplementary material for: Metamaterials for Bone Repair: Design, Mechanisms, and Translation
Source: Biomater Res. 2026 Nov 13;30:0438. doi: 10.34133/bmr.0438 (PMC13760058; doi:10.34133/bmr.0438)
Supplement: Supplementary file 1 [file bmr.0438.f1.docx]

Table 1 summarizes the representative material platforms, architectural features, principal biological roles, limitations, and suitable indications discussed in Sections 3.1 to 3.4.

**Table 1. Comparative summary of material platforms for metamaterials used in bone repair.**

| **Material category** | **Representative systems** | **Principal design features** | **Main biological roles** | **Main limitations and suitable indications** | **Ref.** |
| --- | --- | --- | --- | --- | --- |
| Metallic metamaterials | Ti, Ti6Al4V, Ta, Mg, and Zn systems | Lattice structures, TPMS, graded porosity, and surface functionalization | Load transfer, osseointegration, and bone ingrowth | Fatigue, printing defects, corrosion control, and cost. Suitable for customized load-bearing reconstruction. | [68–69,75,80,90] |
| Bioceramic and bioglass metamaterials | HA, TCP, BCP, mesoporous bioactive glass, and printable bone cements | Hierarchical pores, mineral phase design, ion release, and localized delivery | Mineralization, vascularized bone formation, and local therapeutic delivery | Brittleness, handling strength, and limited long-term load bearing. Suitable for craniofacial, non-load-bearing, and defect-filling applications. | [93,95–96,99,109] |
| Polymeric metamaterials | Silk fibroin, gelatin, alginate, PCL, PLGA, and GelMA | Fibrous networks, directional channels, controlled degradation, and delivery domains | Conformable defect filling and staged biological delivery | Limited standalone strength and space maintenance. Suitable for irregular defects and local delivery. | [115,117,120,127] |
| Composite metamaterials | Metal, ceramic, and polymer combinations with coatings, nanoparticles, or therapeutic payloads | Phase-specific integration of mechanical and biological functions | Coordinated support, mineralization, angiogenesis, immune regulation, and infection control | Manufacturing reproducibility, sterilization, and regulatory burden. Suitable for complex disease-associated defects. | [131,133,136,144,161] |

Fig. 1. Mechanistic roadmap of metamaterial-regulated osteogenesis and vascularization. Scaffold architecture and surface cues activate integrin/FAK/Src and calcium/calcineurin signaling, which interact with YAP/TAZ, Wnt/β-catenin, and BMP-2/Smad pathways to regulate osteogenic gene expression. In parallel, VEGF-A and CD31-associated endothelial responses contribute to vascularization and coordinated bone regeneration.
